# Native low-density lipoproteins are priming signals of the NLRP3 inflammasome/interleukin-1β pathway in human adipose tissue and macrophages

**DOI:** 10.1038/s41598-023-45870-1

**Published:** 2023-11-01

**Authors:** Simon Bissonnette, Valérie Lamantia, Benjamin Ouimet, Yannick Cyr, Marie Devaux, Remi Rabasa-Lhoret, Michel Chrétien, Maya Saleh, May Faraj

**Affiliations:** 1 Faculty of Medicine, Université de Montréal, Montréal, QC Canada; 2 Institut de Recherches Cliniques de Montréal (IRCM), Office 1770.2, 110, Avenue Des Pins Ouest, Montréal, QC H2W 1R7 Canada; 3 Montréal Diabetes Research Center (MDRC), Montréal, QC Canada; 4 Faculty of Medicine, McGill University, Montréal, QC Canada; 5 University of Bordeaux, Bordeaux, France

**Keywords:** Immunology, Biomarkers, Cardiology, Diseases, Endocrinology, Medical research, Molecular medicine, Pathogenesis, Risk factors, Signs and symptoms

## Abstract

Elevated plasma numbers of atherogenic apoB-lipoproteins (apoB), mostly as low-density lipoproteins (LDL), predict diabetes risk by unclear mechanisms. Upregulation of the NLRP3 inflammasome/interleukin-1 beta (IL-1β) system in white adipose tissue (WAT) is implicated in type 2 diabetes (T2D); however, metabolic signals that stimulate it remain unexplored. We hypothesized that (1) subjects with high-apoB have higher WAT IL-1β-secretion than subjects with low-apoB, (2) WAT IL-1β-secretion is associated with T2D risk factors, and (3) LDL prime and/or activate the WAT NLRP3 inflammasome. Forty non-diabetic subjects were assessed for T2D risk factors related to systemic and WAT glucose and fat metabolism. Regulation of the NLRP3 inflammasome was explored using LDL without/with the inflammasome’s priming and activation controls (LPS and ATP). LDL induced *IL1B*-expression and IL-1β-secretion in the presence of ATP in WAT and macrophages. Subjects with high-apoB had higher WAT IL-1β-secretion independently of covariates. The direction of association of LDL-induced WAT IL-1β-secretion to T2D risk factors was consistently pathological in high-apoB subjects only. Adjustment for IL-1β-secretion eliminated the association of plasma apoB with T2D risk factors. In conclusion, subjects with high-apoB have higher WAT IL-1β-secretion that may explain their risk for T2D and may be related to LDL-induced priming of the NLRP3 inflammasome.

ClinicalTrials.gov (NCT04496154): Omega-3 to Reduce Diabetes Risk in Subjects With High Number of Particles That Carry "Bad Cholesterol" in the Blood—Full Text View—ClinicalTrials.gov.

## Introduction

While the role of low-density lipoproteins (LDL) in the pathophysiology of cardiovascular disease is well established, their link to type 2 diabetes (T2D) has only been recently recognized. In 2006, we first proposed that high plasma numbers of apoB-lipoproteins (plasma apoB), more than 90% of which are LDL, may be a promotor and not a mere consequence of T2D as it predicted several plasma pro-inflammatory markers including C-reactive protein and IL-6^1^. We further reported that plasma apoB, but not LDL-cholesterol (LDL-C), predicted white adipose tissue (WAT) dysfunction and related risk factors for T2D including elevated glucose-induced insulin secretion (GIIS), insulin resistance (IR), systemic inflammation, and postprandial hypertriglyceridemia independently of sex and adiposity^1–6^. Moreover, we showed that this may be mediated through an effect on WAT as native LDL inhibited murine^2^ and human^7^ adipocyte differentiation and function as well as human WAT function^2,5^. In line with our findings, epidemiological data emerging since 2007 confirmed that plasma apoB predicts the incidence of diabetes 3–21 years before its onset independently of traditional risk factors including adiposity and glycemia^8–11^. However, mechanisms linking LDL-particles to WAT-related risk factors for T2D remained unexplored.

Activation of the nucleotide-binding domain leucine-rich repeat containing a pyrin domain 3 (NLRP3) inflammasome and IL-1 beta (IL-1β) secretion in immune cells is protective in host defense to infection. However, its chronic activation has been linked to systemic subclinical inflammation, dysfunction of pancreatic beta cells, WAT and muscle and the promotion of T2D^12–16^. The activation of the NLRP3 inflammasome requires 2 signals. The first is a priming signal that is reported to act mostly through the nuclear factor-κB (NF-κB) pathway inducing transcriptional-upregulation of *NLRP3* and mostly *IL1B* and an array of inflammatory cytokines including pro-IL-1β. Priming is also believed to be induced by post-translation modification of NLRP3 independent of its transcription^17–19^. The second signal is an activation signal that leads to the assembly of the NLRP3 inflammasome, activation of pro-caspase-1, cleavage of pro-IL-1β and secretion of IL-1β^12,13^. A wide range of endogenous danger signals, such as oxidized/modified LDL^20,21^, cholesterol crystals^21,22^, glucose^23^, ceramide^15^ and palmitate^24,25^ were described to prime and/or activate the NLRP3 inflammasome in macrophages and β-cells. However, metabolic signals that stimulate the NLRP3 inflammasomes in WAT triggering IL-1β-secretion remain unexplored. Moreover, studies exploring the relation of WAT NLRP3 inflammasome to T2D have focused on the inflammasome priming as *NLRP3* and *IL1B* expressions^15,26,27^, and little to no evidence is garnered in relation to that of its activation as IL-1β-secretion.

We reported in subjects with obesity that plasma apoB predicts plasma IL-1 receptor antagonist (IL-1Ra)^3^, which is a marker of systemic activation of the IL-1 system that precedes the onset of diabetes by 10 years^28^. Statistical adjustment for plasma IL-1Ra eliminated the association of plasma apoB with hyperinsulinemia and IR in this cohort^3^. This suggested that the upregulation of the NLRP3 inflammasome may be a mechanism linking elevated plasma native LDL to the risk of T2D in humans. Thus, we tested the hypotheses that (1) Compared to subjects with low plasma apoB, subjects with high plasma apoB have higher WAT NLRP3 inflammasome activity indicated by higher WAT IL-1β secretion (primary), (2) WAT IL-1β secretion is associated with risk factors for T2D, and (3) native LDL prime and/or activate the NLRP3 inflammasome in subjects’ own WAT ex vivo.

## Materials and methods

### Study design, objectives, and population

This work represents baseline data of a clinical trial that was conducted at the Montréal Clinical Research Institute (IRCM). The trial's central hypothesis was that apoB-lipoproteins act as metabolic danger-associated molecular patterns that activate the NLRP3 inflammasome in WAT leading to WAT dysfunction and associated risks for T2D in humans, which can be treated by eicosapentaenoic and docosahexaenoic acids supplementation. The primary objective examined at baseline was to explore whether subjects with high plasma apoB (high-apoB) have higher WAT NLRP3 inflammasome activity indicated by higher WAT IL-1β secretion than subjects with low plasma apoB (low-apoB). The secondary objectives at baseline were to test whether, (1) WAT IL-1β secretion is associated with risk factors for T2D, and (2) native LDL prime and/or activate the NLRP3 inflammasome in subjects’ own WAT ex vivo. Diabetes risk factors measured were WAT dysfunction, systemic inflammation, postprandial hypertriglyceridemia, GIIS and IR.

Sample size was calculated based on the primary hypothesis/objective and was estimated from post-hoc analysis of 7 subjects with similar characteristics, where plasma apoB correlated with WAT IL-1β-secretion over 4-h (*r* = 0.85, *p* = 0.025). Using average IL-1β-secretion of 437 ± 225 pg/ml/g and assumed power of 80%, ∝ -value of 0.05 and an attrition rate of 20%, N = 20/group were needed to detect an effect size of 1 SD between subjects with high-apoB versus low-apoB.

Volunteers were recruited by advertisement with a similar criteria to a previous trial by our group^29^: males and postmenopausal females, BMI > 20 kg/m^2^, 45–74 years, non-smokers, sedentary, with low/moderate alcohol consumption. Exclusion criteria were elevated cardiovascular risk, chronic disease (i.e. diabetes, inflammatory, autoimmune, hepatic), cancer in the last 3-years, medications affecting metabolism, anemia, abnormal blood coagulation, cholecystectomy, seafood allergy, substance-abuse, sleep-apnea, claustrophobia, and other medical/psychological conditions as judged by the study physicians. Subjects were placed on a 4-week weight-stabilization period verified by weekly weighing (± 2 kg), after which metabolic measures were conducted. The Human Ethics Board of the IRCM approved the research protocol (study number 2013-14). All research was performed in accordance with relevant guidelines/regulations and in accordance with the Declaration of Helsinki. Informed consent was obtained from all participants prior to initiation of any testing. The study was registered retrospectively at ClinicalTrial.org (identifier: NCT04496154) on 03/08/2020. Sample analyses were blinded using subject identification numbers.

### Body composition, energy intake and expenditure

Body composition was assessed by dual-energy Xray absorptiometry (GE Healthcare), basal metabolic rate and substrate oxidation by indirect calorimetry (Vmax Encore; Carefusion), and dietary intake using 3-day-food records and the Food Processor software (V11.3.285, ESHA Research)^2,4,5,7,29,30^.

### Insulin sensitivity and secretion

Subjects followed a 3-day high-carbohydrate diet to maximize glycogen stores after which gold-standard Botnia clamps were conducted as published^3–5,7,29–31^. Briefly, first and second phase GIIS and C-peptide secretion were measured as the AUC of their plasma concentrations during the first 10 min and last 50 min of a 1-h IVGTT, respectively. Insulin sensitivity (IS) was measured during a 3-h hyperinsulinemic euglycemic clamp that followed and expressed as glucose infusion rate divided by steady-state plasma insulin (M/I_clamp_). The disposition index (DI) was calculated as 1st phase or total C-peptide secretion multiplied by M/I_clamp_.

### Postprandial fat metabolism

On a second day of testing, 1–4 weeks after the Botnia clamp, subjects consumed a high-fat meal (600 kcal/m^2^, 68% fat, 18% carbohydrate) after which postprandial plasma clearance of fat and chylomicrons were measured as AUC_6hr_ of plasma TG and apoB48, respectively. Fasting WAT needle-biopsies were collected under local anesthesia (Xylocaine, AstraZeneca) and washed with antibiotic/antifungal supplemented HBSS buffer. One portion was snap-frozen for WAT mRNA and protein expression, and another immediately used for experiments^2,4,5,7,29,30^.

### WAT IL-1β-secretion

Given the absence of studies evaluating the regulation of human WAT NLRP3 inflammasome/IL-1β secretion, pilot kinetic studies were first conducted to establish positive controls for the WAT inflammasome of 4 subjects using LPS as a priming control (Sigma-Aldrich L4591, 0–30 μg/ml, 5 min–24 h) and ATP as an activation control (Sigma-Aldrich A2383, 0–30 mmol/L, 1–20 h) as standard in macrophages^13^. Interleukin-1β secretion from these subjects was not used in data analysis. Minimal concentrations and durations of LPS and ATP that induced maximal WAT IL-1β-secretion were set as positive controls, which corresponded to 0.3 μg/ml LPS for 4 h followed by 3 mmol/L ATP for 3 h (Supplemental Figure 1). Native LDL was isolated from fasting blood samples collected during the Botnia clamp by sequential ultracentrifugation in 0.01% EDTA, sterilized, and used within 4-weeks^2,5,29^. LDL concentration of 1.2 g/L apoB was used, which corresponds to that used to induce human WAT dysfunction^2,5^, the 75th percentile in Canadians^32^, and the average plasma levels in a similar cohort of high-apoB^1,2,29,33^. To assess WAT IL-1β-secretion, WAT samples were incubated for 4 h with medium alone or supplemented with LDL or LPS as priming signals. Medium was removed and WAT was washed and re-incubated for 3 h with medium alone or supplemented with LDL or ATP as activation signals. Medium accumulation of IL-1β was then quantified (termed WAT IL-1β-secretion). All WAT experiments were run in 5% FBS DMEM medium (Gibco/Thermo Fisher) using 5–10 mg WAT/well, 4 wells/condition^2,5,7^. Cell death was verified by a lactate dehydrogenase (LDH) kit (Invitrogen-ThermoFisher Scientific).

**Figure 1:**
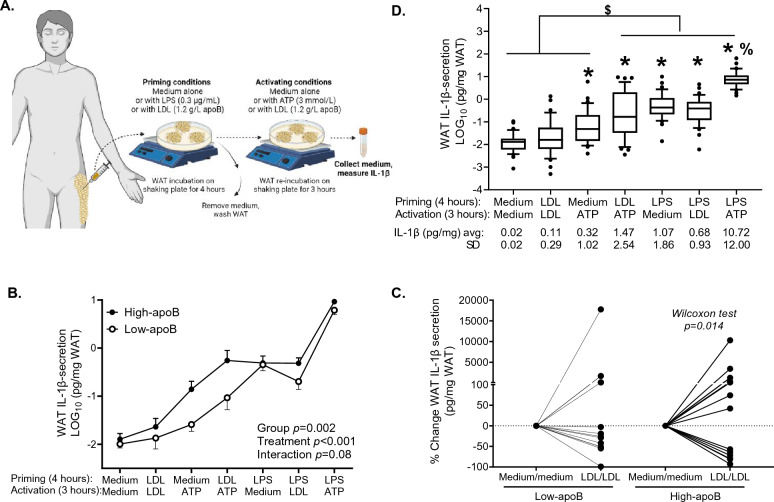
Regulation of WAT IL-1β-secretion ex vivo by LDL, LPS and/or ATP in subjects with high-apoB and low-apoB: experimental design to assess WAT IL-1β-secretion (**A**), WAT IL-1β-secretion induced by the 7-incubation-conditions in all subjects (**B**) WAT IL-1β-secretion in subjects with high-apoB versus subjects with low-apoB (**C**), and % change in WAT IL-1β-secretion induced by LDL/LDL compared to baseline in subjects with low-apoB and high-apoB (**D**). Data was analyzed by mixed-method analyses and presented as boxes with whiskers representing the 10th–90th percentile and a line at the average in panel **B**, as average +/− SEM in panel **C** and by Wilcoxon rank sum test in panel **D**. N = 23 females and N = 11 males except with LDL/LDL where N = 21 females and N = 10 males for panel **B**, and N = 15 for low-apoB and N = 19 for high-apoB except with LDL/LDL where N = 14 for low-apoB and N = 17 for high-apoB for panel **C** and **D** for missing data. *for *p* < 0.001 compared to baseline or LDL/LDL, ^$^for *p* ≤ 0.01 compared to baseline, LDL/LDL, or medium/ATP incubations, and ^%^for *p* < 0.001 compared to all other conditions.

### Experiments in human monocyte-derived-macrophages (hMDM) and differentiated THP-1 macrophages

Blood mononuclear cells (PBMCs) were isolated using Ficoll-Paque Plus (Sigma Aldrich), plated (1 × 10^6^ cells/mL in 24-well plates) and incubated for 2-h with RPMI-1640 medium supplemented with 10% heat-inactivated FBS (Wisent), 1% of penicillin–streptomycin and 2 mM L-Glutamine (Gibco/Thermo Fisher) (baseline medium). Medium was removed and adherent monocytes were differentiated for 6-days in fresh baseline medium with 50 ng/mL macrophage colony-stimulating factor (MCSF, Biolegend)^34^. THP-1 cells (ATCC) were cultured in baseline medium with 0.05 mM 2-mercaptoethanol, and medium was replaced every 2–3 days keeping cell-density between 1 × 10^5^ and 8 × 10^5^. Cells were differentiated for 48-h between passages 15–25 and plated (4 × 10^5^ cells/mL) in baseline medium with 100 nM Phorbol 12-Myristate 13-Acetate (Sigma Aldrich). Macrophages were rested in medium for 48-h before being used^35^. Macrophages were incubated with LDL or LPS (10 ng/mL^34^) and gene expression and IL-1β-secretion were quantified or re-incubated with ATP (3 mmol/L for 3 h) and IL-1β-secretion was quantified.

### mRNA and protein expressions

Gene expression in WAT and macrophages were analyzed by RT-PCR using RotorGene Q (Qiagen) with *HPRT* as an internal standard. WAT proteins were extracted in RIPA buffer and quantified by western blot using an internal control made from pooled WAT from 5 subjects^7,30^. mRNA expression in macrophages was calculated relative to their respective control using the 2^−ΔΔCT^ method. The list of primers and antibodies were previously published^7,30^.

### Plasma and WAT metabolic parameters

Plasma lipids, apoB and apoA1 were analyzed by an automated analyzer (Cobas Integra 400, Roche Diagnostics), glucose by YSI (2300 STAT Plus), apoB48 and PCSK9 (proprotein convertase subtilisin/ kexin type 9) by ELISA kits (BioVendor and CircuLex MBL International), insulin and C-peptide by RIA kits (Millipore Corporation) and IL-1Ra and IL-1β by alpha-LISA kits (Perkin Elmer Canada) and plasma phospholipid-fatty acid composition (PL-FA) by gas-chromatography/mass spectrometry^2,4,5,7,29–31^.

### Statistical analysis

Baseline data in Tables 1 and 2 are presented as mean ± SD and compared by unpaired t-test. Data with large intersubject-variability were LOG_10_-transformed before being used. Non-parametric Friedman or Wilcoxon rank sum tests were used when data could not be transformed (negative or zero values). As WAT and/or LDL from some subjects were insufficient to complete all experiments, mixed-model analyses were used with interaction and Geisser-Greenhouse correction and with controlling for false-discovery rate. When interaction was significant, inter or intra-subject differences were further analyzed. Pearson correlation was used to examine the association between variables in the low-apoB and high-apoB groups separately and data was pooled when no group-differences in the regression lines existed. Partial correlation was used to adjust the association of plasma apoB with diabetes risk factors for covariates. Analysis was performed using SPSS (V26) and GraphPad Prism (V 9.4) with significance set at *p* < 0.05.

**Table 1:** Anthropometric and metabolic parameters of the study population.

	Females (N = 27)	Males (N = 13)	*p *value
Anthropometric parameters			
Age (years)	58.7 ± 7.3	56.0 ± 9.4	0.326
Weight (kg)	76.8 ± 14.5	94.1 ± 20.7	**0.004**
BMI (kg/m^2^)	30.1 ± 5.5	30.4 ± 6.1	0.876
Waist circumference (cm)^a^	95.4 ± 14.8	105.6 ± 15.3	0.051
Hip circumference (cm)	108.3 ± 15.8	108.5 ± 14.1	0.961
Fat mass (kg)	32.9 ± 10.4	29.6 ± 13.1	0.395
Android fat mass (kg)	2.96 ± 1.24	3.06 ± 1.60	0.839
Gynoid fat mass (kg)	5.53 ± 1.56	4.44 ± 2.25	0.082
Android/gynoid ratio	0.52 ± 0.13	0.68 ± 0.15	**0.001**
Fasting metabolic parameters			
SBP (mm Hg)	121 ± 14	122 ± 9	0.775
DBP (mm Hg)	75 ± 8	75 ± 7	0.879
Plasma apoB (g/L)	1.03 ± 0.21	1.05 ± 0.30	0.797
Plasma apoB48 (mg/L)	6.07 ± 2.74	7.39 ± 4.19	0.242
Plasma apoA-I (g/L)	1.72 ± 0.23	1.43 ± 0.20	**< 0.001**
Plasma cholesterol (mmol/L)	5.35 ± 0.81	4.89 ± 0.90	0.111
Plasma LDL-C (mmol/L)	3.18 ± 0.74	3.06 ± 0.77	0.649
Plasma HDL-C (mmol/L)	1.66 ± 0.35	1.13 ± 0.26	**< 0.001**
Plasma TG (mmol/L)	1.11 ± 0.47	1.51 ± 0.93	0.075
Plasma NEFA (mmol/L)	0.633 ± 0.211	0.494 ± 0.244	0.070
Plasma PCSK9 (ng/mL)	227.7 ± 74.4	181.7 ± 56.6	0.056
Plasma apoB/PCSK9 ratio (mg/µg)	4.90 ± 1.64	5.94 ± 1.56	0.065
Plasma IL-1Ra (pg/ml)	473 ± 357	334 ± 189	0.198
Energy intake and expenditure			
Daily energy intake (kcal/day)^b^	2022 ± 426	2689 ± 505	**< 0.001**
% fat intake^b^	37.3 ± 4.9	31.2 ± 6.5	**0.003**
% saturated fat intake^b^	12.2 ± 3.5	10.3 ± 4.8	0.188
Cholesterol intake (mg/day)^a^	310 ± 144	313 ± 162	0.960
% carbohydrate intake^b^	46.3 ± 4.9	50.9 ± 10.2	0.069
Fiber intake (g/day)^b^	21.4 ± 9.4	33.0 ± 13.1	**0.004**
% protein intake^b^	16.5 ± 2.9	16.3 ± 3.4	0.861
% alcohol intake^b^	1.12 ± 1.9	3.51 ± 4.1	**0.019**
Basal metabolic rate (kcal/day)	1337 ± 204	1755 ± 322	**< 0.001**
% carbohydrate oxidation	16.3 ± 12.6	12.5 ± 14.6	0.423
% fat oxidation	63.1 ± 13.8	71.8 ± 13.7	0.082
Botnia clamp measures			
Fasting plasma glucose (mmol/L)	5.11 ± 0.62	4.94 ± 0.31	0.329
Fasting plasma insulin (µU/mL)^c^	11.2 ± 7.7	12.9 ± 9.0	0.535
Fasting plasma C-peptide (ng/ml)^c^	1.76 ± 0.92	1.67 ± 0.73	0.768
HOMA-IR (mmol/L) × (µU/mL)^c^	2.64 ± 2.37	2.90 ± 2.31	0.752
1st phase GIIS_IVGTT_ (µU/mL)^d^	458 ± 380	566 ± 301	0.407
2nd phase GIIS_IVGTT_ (µU/mL)^c^	1703 ± 1529	2268 ± 1943	0.334
Total GIIS_IVGTT_ (µU/mL)^d^	2167 ± 1863	2628 ± 2121	0.510
1st phase C-peptide_IVGTT_ (ng/mL)^d^	34.1 ± 17.5	38.3 ± 10.5	0.466
2nd phase C-peptide_IVGTT_ (ng/mL)^d^	209 ± 112	208 ± 70	0.986
Total C-peptide_IVGTT_ (ng/mL)^d^	243 ± 128	246 ± 79	0.933
M/I_clamp_ (mg/kg*min)/(µU/ml))^c^	0.092 ± 0.048	0.097 ± 0.046	0.756
Fasting WAT IL-1β-secretion and postprandial plasma fat clearance			
Baseline WAT IL-1β-secretion (pg/mg)^e^	0.022 ± 0.028	0.012 ± 0.008	0.220
LPS/ATP-induced WAT IL-1β-secretion (pg/mg)^e^	9.01 ± 4.75	14.29 ± 20.06	0.235
AUC_6h_ plasma TG (mmol/L)	10.9 ± 3.9	16.6 ± 9.1	**0.008**
AUC_6h_ plasma apoB48 (mg/L)	66.5 ± 23.0	77.3 ± 37.3	0.267

Significant values are in bold.

Data are presented as mean ± SD and analyzed using unpaired Student's *t* test.

^a^For N = 26 females.

^b^For N = 25 females and N = 12 males.

^c^For N = 12 males.

^d^For N = 11 males.

^e^For N = 23 females and N = 11 males for missing data.

**Table 2:** Anthropometric and metabolic parameters of subjects with low and high plasma apoB.

	Low-apoB	High-apoB	*p* value
Females: males	13:7	14:6	
Plasma apoB (g/L)	0.84 ± 0.14	1.23 ± 0.15	**< 0.001**
Anthropometric parameters			
Age (years)	56.4 ± 7.5	59.3 ± 8.5	0.268
Weight (kg)	79.3 ± 12.6	85.6 ± 22.7	0.279
BMI (kg/m^2^)	28.7 ± 3.6	31.6 ± 7.0	0.112
Waist circumference (cm)^a^	97.0 ± 9.6	100.7 ± 20.1	0.460
Hip circumference (cm)	106.9 ± 6.6	109.8 ± 20.5	0.558
Fat mass (kg)	28.8 ± 7.4	34.8 ± 13.7	0.093
Android fat mass (kg)	2.59 ± 0.79	3.40 ± 1.66	0.058
Gynoid fat mass (g)	4.77 ± 1.29	5.57 ± 2.26	0.176
Android/gynoid (g/g)	0.55 ± 0.15	0.59 ± 0.16	0.430
Fasting metabolic parameters			
SBP (mm Hg)	119 ± 9	124 ± 14	0.218
DBP (mm Hg)	75 ± 8	76 ± 8	0.741
Plasma apoB48 (mg/L)	5.71 ± 3.28	7.29 ± 3.17	0.128
Plasma apoA-I (g/L)	1.57 ± 0.22	1.68 ± 0.28	0.168
Plasma cholesterol (mmol/L)	4.61 ± 0.55	5.78 ± 0.69	**< 0.001**
Plasma LDL-C (mmol/L)	2.60 ± 0.44	3.68 ± 0.56	**< 0.001**
Plasma HDL-C (mmol/L)	1.56 ± 0.43	1.42 ± 0.38	0.271
Plasma TG (mmol/L)	0.99 ± 0.50	1.50 ± 0.72	**0.013**
Plasma NEFA (mmol/L)	0.599 ± 0.230	0.577 ± 0.233	0.769
Plasma PCSK9 (ng/mL)	212 ± 84	214 ± 59	0.944
Plasma apoB/PCSK9 (mg/µg)	4.42 ± 1.48	6.06 ± 1.45	**0.001**
Plasma LDL-C/apoB (mmol/g)	3.11 ± 0.07	3.00 ± 0.08	0.341
Fasting plasma glucose (mmol/L)	5.09 ± 0.68	5.03 ± 0.38	0.741
Fasting plasma insulin (µU/mL)^b^	11.3 ± 8.3	12.1 ± 8.0	0.745
Fasting plasma C-peptide (ng/ml)^b^	1.58 ± 0.90	1.87 ± 0.80	0.285
HOMA-IR (mmol/L) × (µU/mL)^b^	2.70 ± 2.70	2.74 ± 1.97	0.957
Energy intake and expenditure			
Daily energy intake (kcal/day)^c^	2161 ± 560	2312 ± 539	0.410
% fat intake^c^	32.3 ± 5.0	38.2 ± 5.8	**0.002**
% saturated fat intake^c^	10.1 ± 2.9	13.0 ± 4.4	**0.027**
Cholesterol intake (mg/day)^c^	266 ± 137	354 ± 149	0.070
% carbohydrate intake^c^	51.7 ± 6.9	44.0 ± 5.5	**0.001**
Fiber intake (g/day)^c^	25.5 ± 12.9	24.9 ± 11.1	0.883
% protein intake^c^	16.2 ± 2.2	16.7 ± 3.7	0.616
% alcohol intake^c^	1.46 ± 1.87	2.31 ± 3.70	0.392
Basal metabolic rate (kcal/day)	1418 ± 260	1528 ± 359	0.274
% carbohydrate oxidation	18.4 ± 14.0	12.3 ± 12.1	0.165
% fat oxidation	62.0 ± 13.9	69.2 ± 13.9	0.126

Significant values are in bold.

Data are presented as mean ± SD. Data analyzed using unpaired Student's *t* test.

^a^For N = 13 females in the high-apoB.

^b^For N = 6 males in the low-apoB.

^c^For N = 12 females and N = 6 males in the low-apoB and N = 13 females in the high-apoB groups for missing data.

### Ethics approval and consent to participate

Subjects signed an informed consent form approved by the IRCM Human Ethics Board prior to participation in the study, which was conducted in accordance with the Declaration of Helsinki.

### Consent for publication

Not applicable.

## Results

This report represents the baseline data of a 3-month clinical trial with omega-3 fatty acid supplementation. Subject screening was completed between 2013 and 2019, during which 930 subjects were screened and 27 females and 14 males who met the inclusion/exclusion criteria were enrolled. Common exclusion criteria were related to lack time to participate in all the study, high level of physical activity and plasma metabolic measures outside the inclusion criteria. One man with unreported sleep-apnea was excluded at baseline for safety. The baseline characteristics of the 40 subjects included in this analysis are presented in Table 1. Males had higher weight and central obesity, basal metabolic rate, and energy, fiber and alcohol intake but lower fat intake. Males also had lower fasting plasma HDL-C and apoA1 and delayed postprandial plasma TG clearance.

### Native LDL upregulate IL-1β-secretion from human WAT

To quantify IL-1β-secretion from subjects’ WAT while assessing if and where are native LDL acting in the WAT NLRP3 inflammasome, we assessed priming versus activation using standard positive controls for priming (LPS) and activation (ATP) of the NLRP3 inflammasome in murine and human macrophages^13,36^ (Fig. 1A).

As presented in Fig. 1B, unstimulated baseline WAT IL-1β-secretion (medium/medium for priming/activation) was close to the detection limit (kit = 0.58 ± 0.40 pg/ml vs subject = 0.61 ± 2.50 pg/mL) and maximal induction was attained with the positive controls LPS/ATP that was higher than all other incubation-conditions (10.7 ± 12.0 pg/mg, ~ 1000-fold vs baseline). Stimulating subject WAT with their own LDL (LDL/LDL) increased IL-1β-secretion with large inter-subject variability that did not attain significance. Stimulating WAT with medium/ATP, LDL/ATP, LPS/medium and LPS/LDL significantly increased IL-1β-secretion above baseline and LDL/LDL levels. Stimulating WAT with LDL before activation with ATP (LDL/ATP) increased IL-1β-secretion above that with ATP alone (*p* = 0.015), suggesting that LDL are priming signals of the NLRP3 inflammasome. Conversely, LDL had no effect on IL-1β-secretion when added after LPS compared to LPS alone, suggesting that LDL have no effect on the inflammasome activation. There were no sex-differences in WAT IL-1β-secretion under all incubation-conditions (Supplemental Figure 2). Moreover, LDH release, as a surrogate marker of lytic cell death, was equivalent to baseline under all incubation-conditions indicating that IL-1β-secretion was independent of cell death (Supplemental Figure 3). WAT IL-1β-secretion was correlated with total and central adiposity with LPS stimulation only (Supplemental Figure 4).

### Subjects with high-apoB have higher WAT IL-1β-secretion

To test the primary hypothesis, we separated the 40 subjects following their enrolment around median plasma apoB per sex (Table 2). Average plasma apoB for the low-apoB group (0.84 ± 0.14 g/L) and the high-apoB group (1.23 ± 0.15 g/L) corresponded to < 50th and > 75th percentile in a Canadian population, respectively^32^, similar to values obtained in previous cohorts where the relation of plasma apoB to T2D risk factors was first established^1,2,29^. Subjects with high-apoB had higher fasting plasma total cholesterol, LDL-C, TG and apoB/PCSK9 ratio, higher % total and saturated fat intake and lower % carbohydrate intake. There was no other group-difference including in plasma PL-FA profile, which suggests similar LDL PL-FA profile between the two apoB-groups (Supplemental Figure 5). There were also no significant group-differences in the expression of genes related to WAT differentiation and function (*ADIPOQ, PPARG*), lipid metabolism and sensing (*HMGCR, SREBP1C, SREBP2*), LDL and FA receptors (*LDLR, CD36*), inflammation including *MCP1* (monocyte chemoattractant), *ADGRE1* (macrophage marker), anti-inflammatory *IL10* and the inflammasome-subunits (*NLRP3, CASP1, IL1B*) (Supplemental Figure 6).

As hypothesized, subjects with high-apoB had higher WAT IL-1β-secretion than those with low-apoB (group-effect *p* = 0.002, Fig. 1C), most evident with ATP incubations without/with LDL. Moreover, LDL/LDL induced WAT IL-1β-secretion above baseline in subjects with high-apoB only (Fig. 1D) explaining the large inter-subjects’ variability in Fig. 1B. Statistical adjustment for several covariates related to body composition, lipoproteins, energy intake and expenditure, WAT mRNA, WAT pro-IL-1β, or plasma PL-FA did not eliminate group-differences in WAT IL-1β-secretion (Table 3).

**Table 3:** Differences in WAT IL-1β-secretion induced by the 7-incubation conditions with medium, LDL, LPS and/or ATP in subjects with low-apoB (N = 13) and high-apoB (N = 16) before and after adjustment for covariates.

Adjusted for	Group-effect *p* value
No adjustment	**0.003**
Body composition	
BMI	**0.005**
Total body fat	**0.006**
BMI and waist/hip circumference ratio	**0.007**
Total body fat and android/gynoid ratio	**0.008**
Fasting plasma lipoprotein-related parameters	
Plasma cholesterol	**0.009**
Plasma LDL-C	**0.005**
Plasma HDL-C	**0.003**
Plasma NEFA	**0.004**
LOG_10_ plasma TG	**0.006**
Plasma apoB	0.079
Plasma PCSK9	**0.004**
Plasma apoB/PCSK9	**0.029**
Plasma LDL-C/apoB	**0.006**
Energy intake and expenditure	
Basal metabolic rate	**0.003**
Daily energy intake	**0.001**
% carbohydrate intake	**0.022**
% fat intake	**0.011**
% saturated fat intake	**0.005**
LOG_10_ fasting baseline WAT mRNA	
*PPARG*	**0.005**
*ADIPOQ*	**0.002**
*HMGCR*	**0.005**
*SREBP1C*	**0.004**
*SREBP2*	**0.009**
*CD36*	**0.007**
*LDLR*	**0.004**
*MCP1*	**0.005**
*ADGRE1*	**0.008**
*NLRP3*	**0.007**
*IL1B*	**0.003**
*CASP1*	**0.002**
*IL10*	**0.005**
Fasting baseline WAT protein	
Pro-IL-1β	**0.003**
% plasma phospholipid FA	
% total saturated FA	**0.007**
% total monounsaturated FA	**0.008**
% total omega-3 FA	**0.007**
% total omega-6 FA	**0.009**
% total polyunsaturated FA	**0.009**

Significant values are in bold.

Data analyzed by 2-way RM-ANOVA. N.B. Only subjects with complete WAT IL-1β-secretion profiles induced by the 7-incubation conditions were used in this analysis.

### Native LDL upregulate *IL1B* mRNA and IL-1β-secretion in human primary macrophages

**Figure 2:**
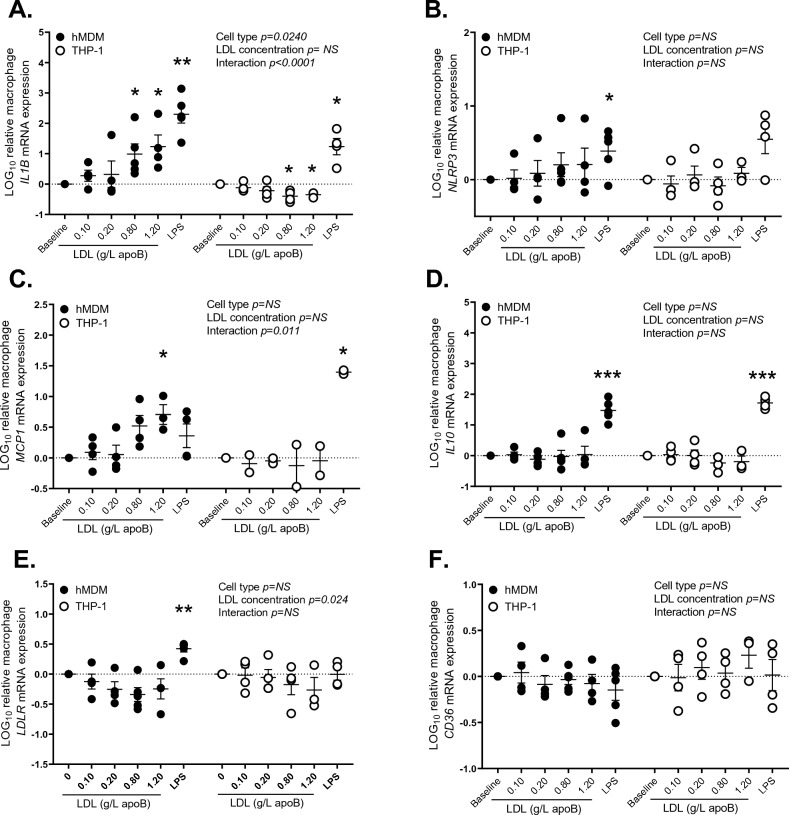
Effects of LDL versus LPS on gene expression in human macrophage models: the effect of native LDL on the mRNA expression of *IL1B* (**A**), *NLRP3* (**B**), *MCP1* (**C**), *IL10* (**D**), *LDLR* (**E**) and *CD36* (**F**) in hMDM and THP-1 derived macrophages using baseline medium supplemented with subject own LDL (0.1–1.2 g/L apoB) or LPS (0.01 μg/mL) over 4 h. Data was analyzed by mixed-method analyses, *for *p* < 0.05 and **for *p* < 0.01 compared to baseline medium alone.

**Figure 3:**
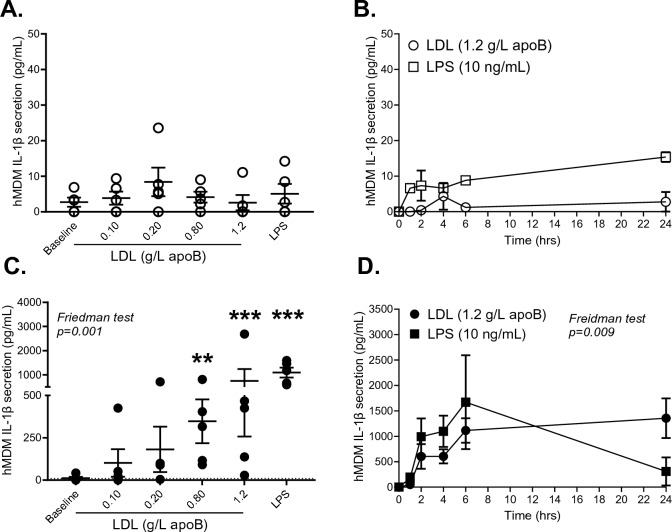
Effects of LDL versus LPS on IL-1β-secretion from hMDM: IL-1β-secretion from hMDM from 5 subjects after incubation with their own native LDL (0.1–1.2 g/L apoB) over 4 h (**A**) or with 1.2 g/L LDL over 1–24 h (**B**) without ATP. Experiments in panel **A** following removal of priming medium and re-incubation with ATP (3 mmol/L) over 3 h (**C**), and experiments in panel **B** following removal of medium and re-incubation with ATP (3 mmol/L) over 3 h (**D**). LPS (10 ng/mL) was used as a priming control in all experiments. Data was analyzed by non-parametric Friedman test separately for LDL. **for *p* < 0.01 and ***for *p* < 0.001 versus baseline.

As the yield from WAT needle-biopsies limited the number of experiments that can be conducted, we further examined the effects of LDL on the inflammasome priming in hMDM over 4 h as conducted in WAT. hMDM were used given the detection of macrophage marker *ADGRE1* in subject WAT and that we did not detect *NLRP3* expression in a human model of primary adipocytes^7^. As presented in Fig. 2A, similar to LPS, LDL increased *IL1B* expression in a concentration-dependant manner in hMDM. However, unlike LPS, the same LDL preparation decreased *IL1B* expression in THP-1- macrophages, suggesting that this cell-line may not be a suitable model of unpolarized hMDM. LDL also increased *MCP1* expression in hMDM (Fig. 2C) but had no significant effect on *NLRP3, IL10,* or *LDLR*, while both LDL and LPS had no affect on *CD36* expression in either macrophage-model (Fig. 2B, D–F). To explore if LDL-induced increase in *IL1B* expression translates into IL-1β-secretion, IL-1β-secretion before and after activation of the NLRP3 inflammasome by ATP was measured. Neither LDL nor LPS alone triggered IL-1β-secretion in hMDM using various LDL-concentrations (Fig. 3A) and up to 24 h without ATP (Fig. 3B). However, when followed by ATP, LDL induced IL-1β-secretion in a dose- (Fig. 3C) and time-dependent manner (Fig. 3D). LDL did not induce IL-1β-secretion from THP-1-derived macrophages without or with ATP (data not shown).

### WAT IL-1β secretion are consistently associated with higher diabetes risk factors in subjects with high-apoB

**Figure 4:**
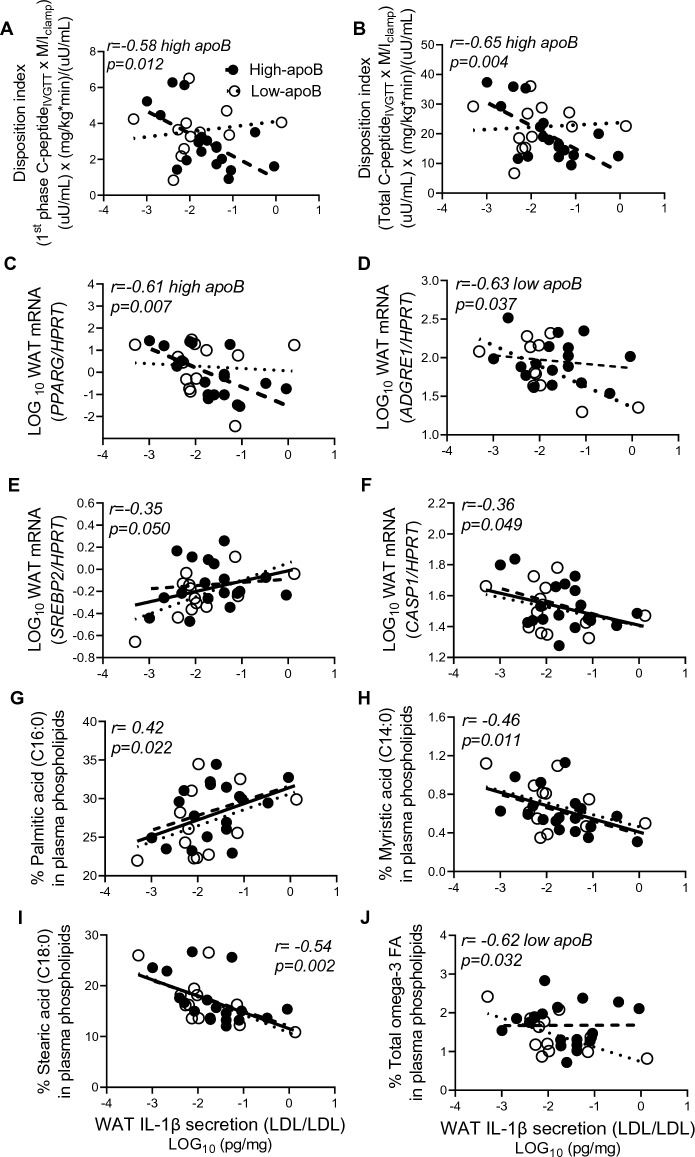
Correlations of LDL/LDL-induced WAT IL-1β-secretion with diabetes risk factors and % fasting plasma PL-FAs: Correlation of WAT IL-1β-secretion with 1st phase disposition index (**A**), total disposition index (**B**), WAT *PPARG* mRNA (**C**), WAT *ADGRE1* mRNA (**D**), WAT *SREBP2* mRNA (**E**), and WAT *CASP1* mRNA (**F**) normalized for *HPRT,* and % plasma PL-palmitic acid (**G**), myristic acid (**H**), stearic acid (**I**), and total omega-3 FA (**J**) in subjects with low-apoB (N = 14) and high-apoB (N = 17). Solid regression line represents correlation in all subjects.

Confirming our previous findings in a similar cohort^2–5,29^, plasma apoB was associated positively with measures of GIIS_IVGTT_, IR, plasma IL-1Ra, postprandial hypertriglyceridemia and hyperchylomicronemia (Supplemental Figure 7). Here, we tested whether WAT IL-1β-secretion was associated with these risk factors. Indeed, LDL-induced WAT IL-1β-secretion was associated to metabolic risk in the high-apoB group as it was inversely correlated with 1^st^ phase and total DI, which is a predictor of diabetes risk^37^ and with WAT *PPARG*, which is a marker of adipocyte differentiation (Fig. 4A–C). Conversely, these associations were absent in the low-apoB group, in whom IL-1β-secretion was inversely related to macrophage infiltration (Fig. 4D). In both groups, LDL-induced IL-1β-secretion was positively associated with WAT *SREBP2,* which is upregulated upon NLRP3 inflammasome activation^13,38^, and negatively with *CASP1,* which is required for adipocyte differentiation^14^ (Fig. 4E, F). Importantly, the profile of LDL-FA appears to modulate WAT IL-1β-secretion, as higher palmitate but lower myristate and stearate were associated with higher IL-1β-secretion in both groups, while higher omega-3 associated with lower IL-1β-secretion in the low-apoB group (Fig. 4G–J).

**Figure 5:**
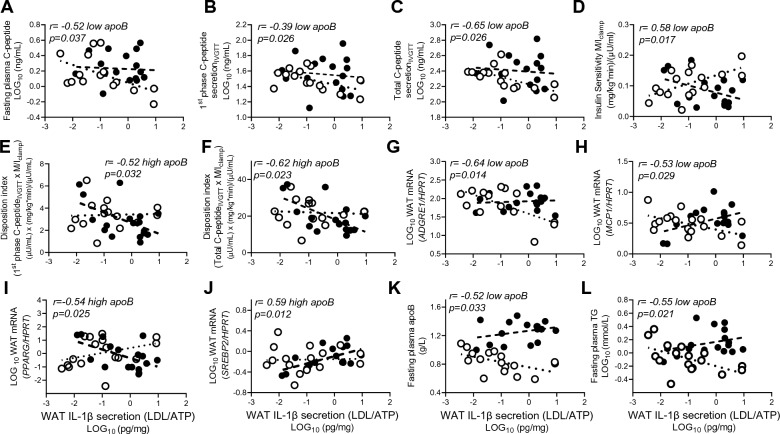
Correlations of LDL/ATP-induced WAT IL-1β-secretion with diabetes risk factors: Correlation of WAT IL-1β-secretion with fasting plasma C-peptide (**A**), 1st phase C-peptide secretion_IVGTT_ (**B**), total C-peptide secretion_IVGTT,_ (**C**), insulin sensitivity as M/I_clamp_ (**D**), 1st phase disposition index (**E**), total disposition index (**F**), WAT *ADGRE1* mRNA (**G**), WAT *MCP1* mRNA (**H**), WAT *PPARG* mRNA (**I**), and WAT *SREBP2* mRNA (**J**) normalized for *HPRT*, and fasting plasma apoB (**K**) and TG (**L**) in subjects with low-apoB (N = 15) and high-apoB (N = 19). Solid regression line represents correlation in all subjects.

**Figure 6:**
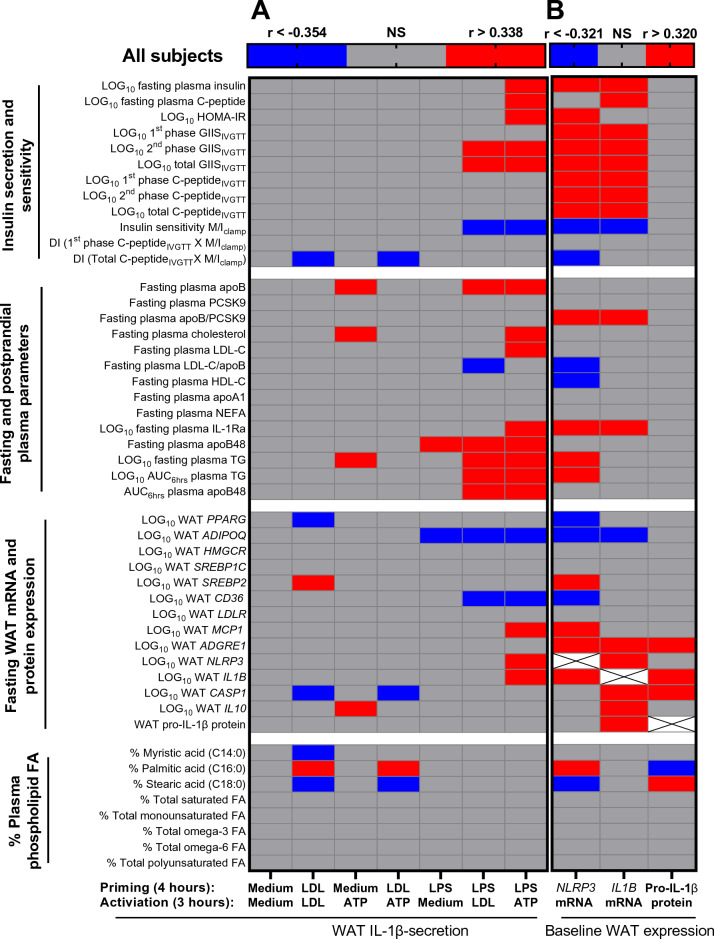
Heat-map representing correlations of WAT NLRP3 inflammasome priming and activation measures with outcome measures: Correlations of WAT IL-1β-secretions induced by the 7-incubation conditions (**A**) and of WAT *NLRP3* mRNA, WAT *IL1B* mRNA, and WAT pro-IL-1β protein (**B**) with fasting and Botnia clamp measures of insulin sensitivity and secretion, fasting and postprandial plasma lipoprotein-related parameters, fasting baseline WAT mRNA expression of genes related to WAT function and inflammation, and % plasma PL-FA in all subjects. Grey cells represent insignificant, blue cells represent significant negative associations, and red cells represent significant positive associations.

Similarly, LDL/ATP-induced WAT IL-1β-secretion was related to metabolic health in the low-apoB group as it associated inversely with fasting plasma C-peptide and glucose-induced 1st phase and total C-peptide secretion_IVGTT_ and positively with IS (Fig. 5A–D). Conversely in high-apoB, WAT IL-1β remained inversely associated with 1st phase and total DI (Fig. 5E, F). WAT IL-1β-secretion was also inversely associated with WAT inflammation in the low-apoB group (negatively with *ADGRE1* and *MCP1*) but with WAT dysfunction in the high-apoB group (negatively with *PPARG* and positively with *SREBP2*) (Fig. 5G–J). Interestingly in the group with low-apoB, the lower was plasma apoB and TG the higher was IL-1β-secretion induced by LDL, suggesting higher sensitivity to LDL effects (Fig. 5K–L). Notably, the contradictory associations of WAT IL-1β-secretion with diabetes risk factors in the low-apoB and high-apoB groups cancelled each other when all subjects were combined (Fig. 6A). Thus, the physiological and diabetogenic associations of WAT IL-1β-secretion would have been missed.

On the other hand, higher LPS-induced IL-1β-secretion was associated with diabetes risk factors without a group-differences. As presented in Fig. 6A and Supplemental Figure 8, WAT IL-1β-secretion, particularly when maximized by LPS/ATP, was associated with higher fasting and clamp-measures of insulin secretion and resistance, delayed fasting and postprandial plasma clearance of TG and apoB48, higher plasma apoB, total and LDL-C and/or IL-1Ra, lower LDL size (estimated from LDL-C/apoB ratio), and/or with markers of WAT dysfunction (inversely with *ADIPOQ* and *CD36)* and WAT inflammation (positively with *MCP1*, *NLRP3* and *IL1B*).

### WAT *NLRP3 and IL1B* mRNA and pro-IL-1β protein are associated with diabetes risk factors in all subjects

Unlike WAT IL-1β-secretion, the expression of WAT *NLRP3* and to a lesser extent *IL1B* was positively associated with all diabetes risk factors examined with no group-differences (Fig. 6B, Supplemental Figure 9 ). Moreover, it was associated with higher plasma apoB/PCSK9 ratio, which is a predictor of WAT surface-expression of LDLR and^7^ and lower estimated LDL size and plasma HDL-C. It was also associated with a proinflammatory plasma PL-FA profile (positively with palmitate and arachidonate and negatively with stearate) and markers of WAT dysfunction/inflammation (inversely with *PPARG, ADIPOQ, CD36* and positively with *SREBP2*, *MCP1*, *ADGRE1, IL1B).* WAT pro-IL-1β protein was also positively associated with WAT *ADGRE1, IL1B* and *CASP1* but inversely with palmitate, suggesting higher cleavage of pro-IL-1β to active IL-1β with this inflammatory plasma PL-FA profile.

### Statistical adjustment for WAT IL-1β-secretion attenuates the association of plasma apoB with diabetes risk factors

To explore whether the WAT NLRP3 inflammasome/IL-1β pathway is related to higher diabetes risk factors in subjects with high-apoB, we used partial correlation analysis. Adjusting for WAT IL-1β-secretion induced by LDL, LPS and/or ATP gradually eliminated the association of plasma apoB with measures of insulin secretion and resistance, plasma IL-1Ra and delayed postprandial chylomicron clearance reaching a maximum with LPS/ATP (Table 4). Adjusting for WAT *NLRP3*, WAT *IL1B* or total and central adiposity had less effects than maximal LPS/ATP-induced IL-1β-secretion, while adjusting for WAT pro-IL-1β protein or waist/hip ratio had little to no effect (Table 4).

**Table 4:** Partial correlation of plasma apoB with risk factors for type 2 diabetes before and after adjustment for LOG_10_ fasting WAT IL-1β-secretion induced by the 7-incubation conditions with medium, LDL, LPS and/or ATP, WAT *NLRP3* mRNA, WAT *IL1B* mRNA and WAT pro-IL-1β protein expression, or body composition measures.

Adjustment for	LOG_10_ 2nd phase GIIS_IVGTT_	LOG_10_ 2nd phase C- peptide secretion_IVGTT_	LOG_10_ total C-peptide secretion_IVGTT_	M/I_clamp_	LOG_10_ plasma IL-1Ra	LOG_10_ AUC_6hrs_ TG	AUC_6hrs_ apoB48
	*p* values						
None	0.038	0.025	0.031	0.001	0.032	< 0.001	0.029
WAT IL-1β (LDL/LDL)	–	0.024	0.029	0.006	–	< 0.001	0.042
WAT IL-1β (medium/ATP)	–	–	–	0.007	0.040	< 0.001	0.031
WAT IL-1β (LDL/ATP)	0.044	0.014	0.018	0.003	–	< 0.001	0.033
WAT IL-1β (LPS/medium)	–	0.043	–	0.004	–	< 0.001	–
WAT IL-1β (LPS/LDL)	–	–	–	0.031	–	0.002	–
WAT IL-1β (LPS/ATP)	–	–	–	–	–	0.002	–
WAT *NLRP3* mRNA	–	–	–	0.010	–	< 0.001	–
WAT *IL1B* mRNA	–	–	–	0.004	–	< 0.001	0.036
WAT pro-IL-1β protein	0.036	0.033	0.041	0.002	0.035	< 0.001	0.025
BMI	–	–	–	0.018	–	< 0.00	0.044
Total fat mass	–	–	–	0.047	–	< 0.001	0.042
Waist/hip ratio	–	0.034	0.043	0.003	0.043	< 0.001	–
Android/gynoid fat ratio	–	–	–	0.012	–	< 0.001	–

N = 27 females and N = 13 males for WAT mRNA and protein and anthropometric parameters, N = 23 females and N = 11 males for WAT IL-1β-secretion conditions except for LDL/LDL condition where and N = 21 females and N = 10 males for missing data.

## Discussion

This study uncovered novel findings regarding the regulation of the human WAT NLRP3 inflammasome/IL-1β pathway by native LDL. We showed that native LDL are metabolic stimuli of the NLRP3 inflammasome in human WAT (Fig. 1) and primary macrophages (Figs. 2, 3) by acting as priming signals inducing *IL1B* expression and IL-1β-secretion in the presence of an activation signal. Accordingly, subjects with high-apoB have higher WAT IL-1β-secretion than those with low-apoB (Fig. 1C) independently of anthropometric and metabolic covariates (Table 3). While WAT *NLRP3* and *IL1B* mRNA expressions were associated with all examined risk factors for T2D without apoB-group differences (Fig. 6B, Supplemental Figure 9), there was a dichotomy in the association of WAT IL-1β-secretion to these factors dependent on the WAT stimuli, IL-1β-secretion levels, and the study group. Higher WAT IL-1β-secretion induced by metabolic stimuli (LDL ± ATP) were positively associated with risk factors for T2D in subjects with high-apoB, while an inverse association was revealed with lower WAT IL-1β-secretion in subjects with low-apoB (Figs. 4, 5). On the other hand, replacing priming with LDL by microbial LPS induced IL-1β-secretion in subjects with low-apoB to levels similar to those in the high-apoB and revealed a diabetological relationship in all subjects, particularly with maximal LPS/ATP-induced stimulation (Fig. 6A, Supplemental Figure 8). It is important to underscore that these results were generated in a healthy population without chronic or infectious disease in an attempt to dissect out LDL-effects in vivo and ex vivo. Whether these findings translate to other populations needs to be examined.

To the best of our knowledge, only one human study examined 1L-1β-secretion from unstimulated subcutaneous WAT, where very low secretion levels were also reported^16^, while none examined its regulation in any human fat depot. Here we report that native LDL, LPS or ATP alone induce IL-1β-secretion from human WAT (Fig. 1). As our findings in hMDM confirm that, for IL-1β to be secreted, 2 signals for priming and activation of the NLRP3 inflammasome are needed (Figs. 2, 3), the induction of IL-1β-secretion from WAT by LDL, LPS or ATP alone implies that an endogenous WAT-released signal(s) must have provided the other missing signal. This signal is likely palmitate, given its release by endogenous lipolysis^39^ and effects on the inflammasome priming and activating in macrophages^24,25^. Endogenous ATP release, which increases with IR in WAT and would further stimulate lipolysis^40^, may have also provided an activation signal, especially in subjects with high-apoB and higher IR. While alone this endogenous signal(s) was insufficient to induce WAT IL-1β-secretion at baseline to detectable levels (Fig. 1), its effects together with the mass of WAT cannot be ignored when evaluating the physiological effects of LDL, ATP and LPS particularly with obesity and IR. Moreover, IL-1β-secretion was independent of cell-death (Supplemental Figure 3). This is in line with recent data demonstrating that IL-1β maturation by caspase-1 triggers its transport to the plasma membrane for both Gasdermin-dependent and -independent release in live cells^41^. The secretion of IL-1β is also proposed to occur in a continuum with secretion-levels depending on the stimulus strength (i.e. type, dose and duration) and extracellular IL-1β requirements^36^.

Many lipoprotein-related components and byproducts mostly studied in macrophages using oxidized or modified LDL in the context of atherosclerosis have been described to regulate the NLRP3 inflammasome. Accumulation of free cholesterol, PL and palmitate was shown to induce lysosomal dysfunction and the leakage of its content (e.g. Ca^2+^ and cathepsin B) that prime and/or activate the NLRP3 inflammasome in macrophages and tubular endothelial cells^25,38,42,43^. Moreover, fatty acid β-oxidation in the mitochondria and/or ceramide synthesis in the ER increase the production of reactive oxygen species that prime and activate the inflammasome^44,45^. More recently, ceramide^46^ and diacyl glycerol^17^, were shown to induce post-translational phosphorylation of NLRP3 needed for subsequent inflammasome activation^17,19,46^. Post-translational modification of NLRP3 may also explain significant induction of *IL1B* expression by native LDL in the absence of an effect on *NLRP3* expression in this study (Fig. 2A, B). Alternatively, the upregulation of *NLRP3* expression may have preceded that of *IL1B* as previously demonstrated in MCSF-differentiated hMDM stimulated by LPS alone or with cholesterol crystals (peaking by 1 h vs 3–6 h, respectively)^22^.

Moreover, intersubject variation in LDL quality may have translated into the observed variations in WAT and macrophage responses to LDL (Figs. 1, 2, 3). While native LDL was used without in vitro modification, the presence of variable content of oxLDL^47^, LDL-bound LPS^48^ or LDL-ceramide^49^ in subject LDL preparations cannot be excluded. These components are measurable in the plasma of subjects without chronic disease and would increase the priming and/or activation of the NLRP3 inflammasome. Here we extend these findings to report that smaller LDL size, higher LDL-enrichment with palmitate and arachidonate and lower enrichment with myristate, stearate and omega-3 FA (in low-apoB) are associated with higher expression of WAT *NLRP3*, depletion of pro-IL-1β and/or secretion of IL-1β (Figs. 4G–J, 6, Supplemental Figure 9S-U). Smaller denser LDL characterize subjects with high-apoB as previously reported using gel electrophoresis^2,3^. Moreover, we reported that normo-cholesterolemic subjects with lower plasma PCSK9 and higher WAT surface-expression of LDLR and CD36 have higher WAT IL-1β-secretion and risk factors for T2D^7^. Thus, receptor-mediated uptake of the whole LDL-particle together with all its components may be needed to mediate the effect of LDL reported here. And while counter-regulatory mechanisms exist to block excessive LDL-uptake via LDLR, such as by inhibiting SREBP2, the activation of the NLRP3 inflammasome in immune cells was shown to activate SREBP2, which in turn can act in a positive feed-back loop promoting further inflammation and cholesterol accumulation^38^. Consistently, there was a strong association between WAT *NLRP3* mRNA (Fig. 6B) or IL-1β-secretion (Figs. 4E, 5J, 6) with WAT *SREBP2* mRNA especially in subjects with high-apoB. Moreover, unlike previously reported temporal effects of LPS^50^ and as shown here, LDL-induced IL-1β-secretion from hMDM remained significant even up to 24-h (Fig. 3D). The intracellular mechanisms regulated by native LDL and their receptors, LDLR and CD36, are further being investigated in an ongoing study on WAT NLRP3 inflammasome (https://classic.clinicaltrials.gov/ct2/show/NCT04485871).

Adjusting for WAT *NLRP3* and *IL1B* mRNA and mostly for maximal LPS/ATP-induced IL-1β-secretion attenuated the association of plasma apoB with diabetes risk factors (Table 4). This supports that WAT NLRP3 inflammasome/IL-1β pathway is a mechanism linking hyperapoB to diabetes risk, however; it does not exclude other mechanisms. We previously reported that LDL decrease the differentiation of a human model of adipocyte in an *NLRP3-*independent manner^7^. Moreover, other groups have reported that receptor-mediated uptake of apoB-lipoproteins in myocytes^51^ and β-cells^52^ induces their metabolic dysfunction.

**Figure 7:**
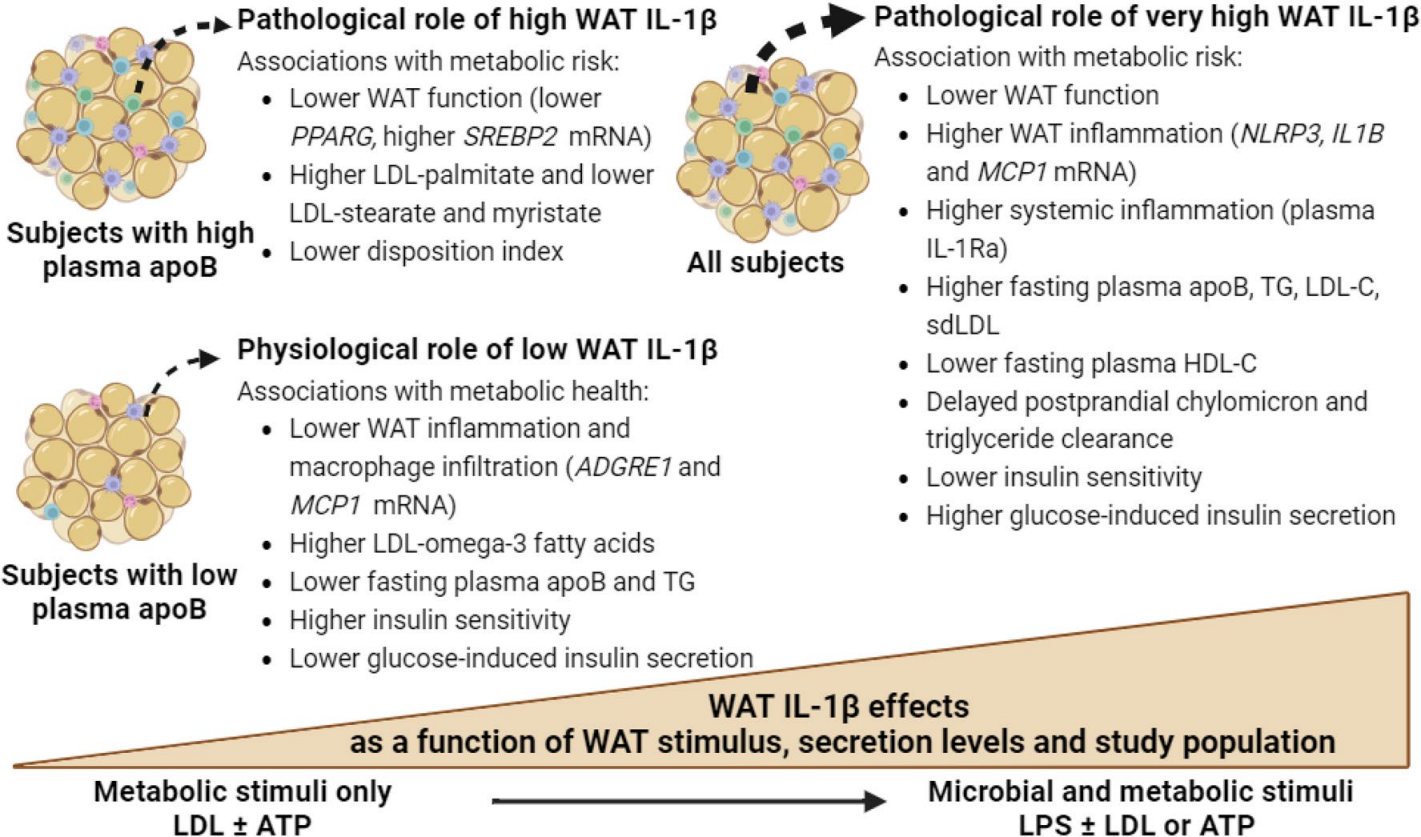
Proposed pathological and physiological roles of WAT IL-1β as a function of WAT stimulus, IL-1β secretion levels, and metabolic health of the study population: In the presence of metabolic stimuli such as LDL ± ATP, higher WAT IL-1β-secretion in subjects with high-apoB has a diabetological pro-inflammatory effect while lower WAT IL-1β-secretion in subjects with low-apoB has a physiological hypoglycemic effect. Microbial stimuli such as LPS maximize the WAT IL-1β-secretion overriding the metabolic effects of IL-1β and unraveling its pathological pro-inflammatory effects and associations with T2D risk factors in all subjects.

Intriguingly, there was a dichotomy in the association of WAT IL-1β-secretion to diabetes risk factors depending on the apoB-group and WAT stimulus (Figs. 4, 5). Conflicting outcomes were also reported regarding targeting the IL-1β pathway in humans. Interleukin-1Ra^53^ or anti-IL-1β^54^ were demonstrated to improve plasma glucose, *β*-cell function, IR, and/or inflammation in diabetic patients. Conversely anti-IL-1β therapy did not decrease diabetes incidence in cardiovascular patients on statins in the CANTOS trial^55^. This led to the proposal that targeting the IL-1β-pathway may reduce diabetes complications rather than prevent its incidence^56^. However, several lines of in vivo and ex vivo evidence indicate that IL-1β has hypoglycemic actions that are independent of insulin action and of IL-1β pro-inflammatory and pyroptotic effects (for review^57^). In vitro, IL-1β was reported to increase glucose-uptake in several cell type including adipocytes, while intraperitoneal injection of nanogram amounts into mice was shown to deplete hepatic glycogen, inhibits gluconeogenesis and reduces plasma glucose by 50% for over 24-h^57^. These physiological and pathological effects of IL-1β are dependent on its amount, and can be overridden with overproduction such as in infectious disease where IL-1β is believed to induce IR in non-immune tissue to deviate glucose-utilization to the immune cells^57^. Thus, taking together published data together with our findings suggest that under non-infectious conditions, WAT IL-1β-secretion may not be pathological in all subjects and should not be targeted in all subjects. The effects of WAT IL-1β may be dependent on the stimulus inducing its secretion, IL-1β secretion levels, and the metabolic health of the population examined encompassing the quality of LDL and WAT (Fig. 7).

In conclusion, we report novel findings that native LDL are priming signals of the human WAT and macrophage NLRP3 inflammasome leading to IL-1β-secretion in the presence of an activation signal. Accordingly, subjects with high plasma apoB have higher WAT IL-1β-secretion, which is associated with higher risk factors for T2D. We propose that subjects with high-apoB may be an ideal population to target the IL-1β pathway given higher WAT IL-1β-secretion in response to both metabolic (LDL and ATP) and microbial (LPS) stimuli and higher risk for T2D in this population.

### Supplementary Information

Supplementary Information.

## Data Availability

The datasets generated during and/or analyzed during the current study are not publicly available due to ethical restrictions but are available from the corresponding author upon reasonable request.

## Abbreviations

ADGRE1: Adhesion G protein-coupled receptor E1
Apo: Apolipoprotein
ATP: Adenosine triphosphate
AUC6hrs: Area under the 6-h postprandial curve
BMI: Body mass index
BSA: Bovine serum albumin
CASP1: Caspase-1
CD36: Cluster of differentiation 36
CTL: Control
DI: Disposition index
EDTA: Ethylenediaminetetraacetic acid
FA: Fatty acids
GIIS: Glucose-induced insulin secretion
HBSS: Hanks’ balanced salt solution
HDL-C: High-density lipoprotein cholesterol
HMGCR: 3-Hydroxy-3-methylglutaryl-CoA reductase
HPRT: Hypoxanthine phosphoribosyltransferase 1
IL-1β: Interleukin 1 beta
IL-1Ra: Interleukin 1 receptor antagonist
IS: Insulin sensitivity
IR: Insulin resistance
IVGTT: Intravenous glucose tolerance test
LDL: Low-density lipoprotein
LDLR: Low-density lipoprotein receptor
LPS: Lipopolysaccharide
MCP-1: Monocyte chemoattractant protein 1
MCSF: Macrophage colony-stimulating factor
M/I: Glucose infusion rate divided plasma insulin at steady state
NEFA: Non-esterified fatty acids
NF-κB: Nuclear factor-κB
NLRP3: Nucleotide-binding domain leucine-rich repeat containing a pyrin domain 3
PBMC: Peripheral blood mononuclear cells
PCSK9: Proprotein convertase subtilisin-kexin type 9
PL: Phospholipids
RT-PCR: Real time PCR
SREBP: Sterol regulatory element binding protein
T2D: Type 2 diabetes
TG: Triglyceride
WAT: White adipose tissue
