# Supplementary material for: Native low-density lipoproteins are priming signals of the NLRP3 inflammasome/interleukin-1β pathway in human adipose tissue and macrophages
Source: Sci Rep. 2023 Nov 1;13:18848. doi: 10.1038/s41598-023-45870-1 (PMC10620147; doi:10.1038/s41598-023-45870-1)
Supplement: Supplementary file 1 — Supplementary Information. [file 41598_2023_45870_MOESM1_ESM.pdf]

Native low-density lipoproteins are priming signals of the NLRP3 inflammasome/ interleukin-1β pathway in human adipose tissue and macrophages

Simon Bissonnette[1,2,3], Valérie Lamantia[1,2,3], Benjamin Ouimet[1,2], Yannick Cyr[1,2,3], Marie Devaux[1,2], Remi Rabasa-Lhoret[1,2,3], Michel Chrétien[1, 2], Maya Saleh[4,5], May Faraj[1,2,3,4]

[1] Faculty of Medicine, Université de Montréal, Montréal, Québec, [2] Institut de recherches cliniques de Montréal (IRCM), Montréal, Québec, [3] Montreal Diabetes Research Center (MDRC), Montréal, Québec, Ottawa, Ontario, [4]Faculty of Medicine, McGill University, Montréal, Québec, [5]University of Bordeaux, Bordeaux, France

Supplemental Figure 1: Pilot kinetic studies conducted in fasting white adipose tissue (WAT) of 4 subjects to optimize experimental conditions to be used for LPS and ATP as *priming* and *activation* positive controls of WAT NLRP3 inflammasome, respectively. Shown here is WAT IL-1β-secretion following incubation with (**A**) LPS concentration-curve for 6 hours followed by 5 mmol/L ATP for 20 min, N=1; (**B**) 1 µg/mL LPS time-curve followed by 5 mmol/L ATP for 20 min, N=1; (**C**) 0.3 µg/ml LPS for 6 hours followed by ATP concentration-curve for 20 minutes, N=3; and (**D**) 0.3 µg/ml LPS for 4 hours followed by 3 mmol/L ATP time-curve, N=2. Minimal LPS and ATP concentrations and incubation period that induced maximal WAT IL-1β-secretion were then used as positive controls for all experiments described in manuscript and were: **0.3 µg/ml LPS for 4 hours followed by 3 mmol/L ATP for 3 hours** (highlighted in red rectangular below). N.B. LPS was used for 4 hours for practical reasons to account for the 8 hours needed to complete experiments in postprandial WAT (not included in this manuscript).

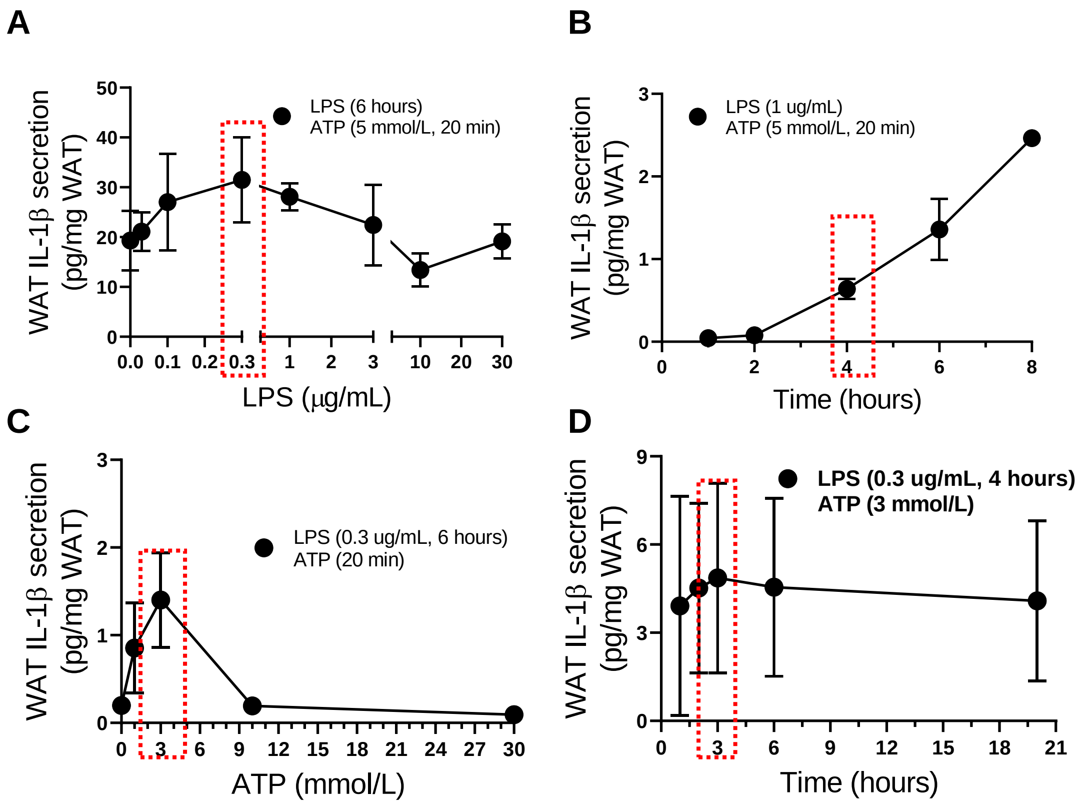

Supplemental Figure 2: Sex differences in fasting WAT IL-1β-secretion induced by 5% FBS medium/medium (baseline or negative control), LDL/LDL, medium/ATP, LDL/ATP, LPS/medium, LPS/LDL, or LPS/ATP (positive control) for the priming/activation periods of the NLRP3 inflammasome. Data was analyzed by mixed-model analysis with treatment and sex interaction as in methods and presented as average +/- SEM. N=23 women and N=11 men except with LDL/LDL, where N=21 women and N=11 men for missing data.

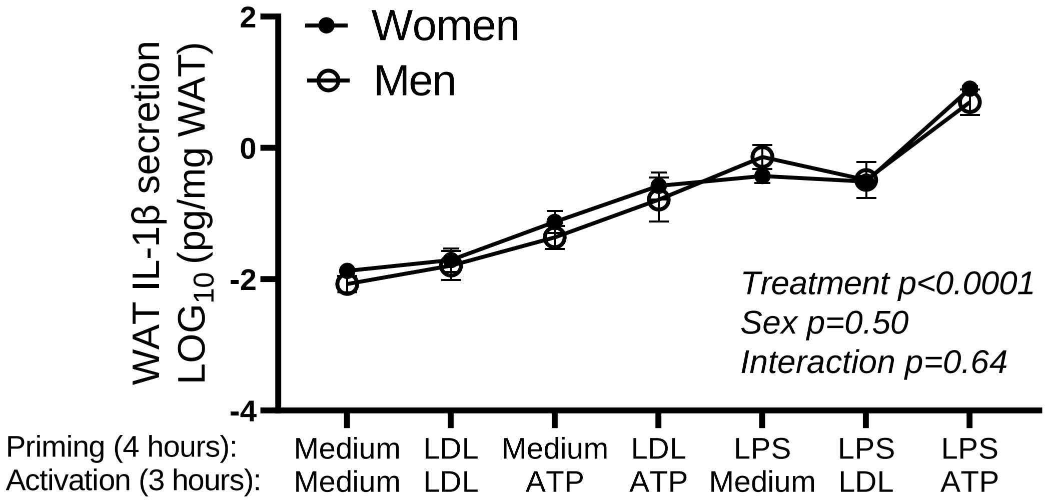

Supplemental Figure 3: Cytotoxic assay measuring LDH release into WAT medium induced by 5% FBS medium/medium (baseline or negative control), LDL/LDL, medium/ATP, LDL/ATP, LPS/medium, LPS/LDL, or LPS/ATP (positive control) for the priming/activation periods of the NLRP3 inflammasome. Also shown are LDH concentrations measured in WAT-free medium alone or suplemented with 1.2 g/L apoB incubated for 3 hours compared to the LDH positive control provided by the commercial kit (Invitrogen). Notably, LDL presence alone (without WAT) represent a modest false positive signal for LDH measurement above baseline (medium).

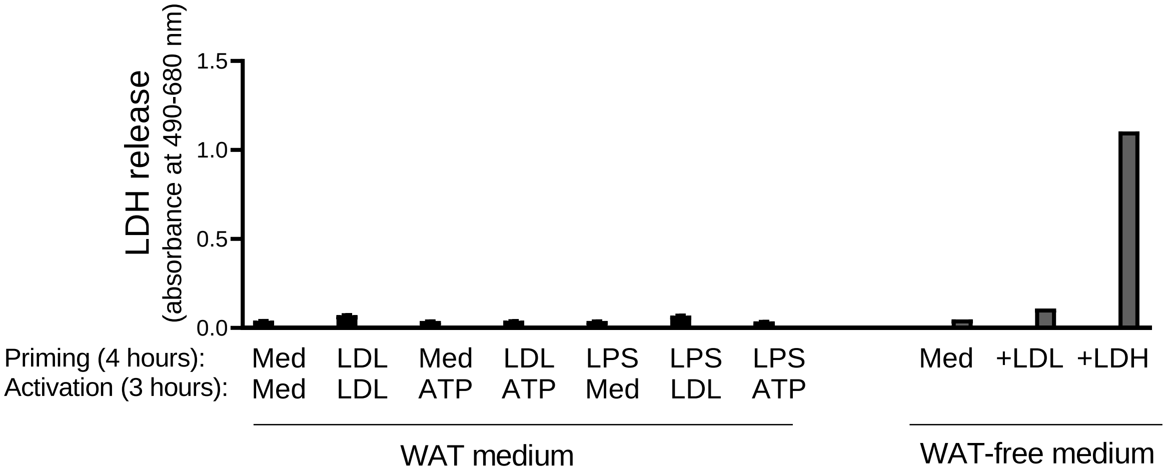

Supplemental Figure 4: Heat map representing Pearson correlation of subject weight, BMI, waist circumference, hip circumference, waist/hip ratio, android fat, gynoid fat, total body fat, android/gynoid fat ratio with fasting LOG_{10} WAT IL-1β-secretion at baseline (medium/medium) and following incubation with LDL/LDL, medium/ATP, LDL/ATP, LPS/medium, LPS/LDL, or LPS/ATP for the priming/activation periods, and with LOG_{10}WAT *NLRP3*, LOG_{10}WAT *IL1B* mRNA and WAT pro-IL-1β protein in all subjects. N=40 for WAT mRNA and pro-IL-1β data and N=34 for all WAT IL-1β-secretion conditions except with LDL/LDL when N=31 for missing data. Grey cells represent insignificant and red cells represent significant positive correlations.

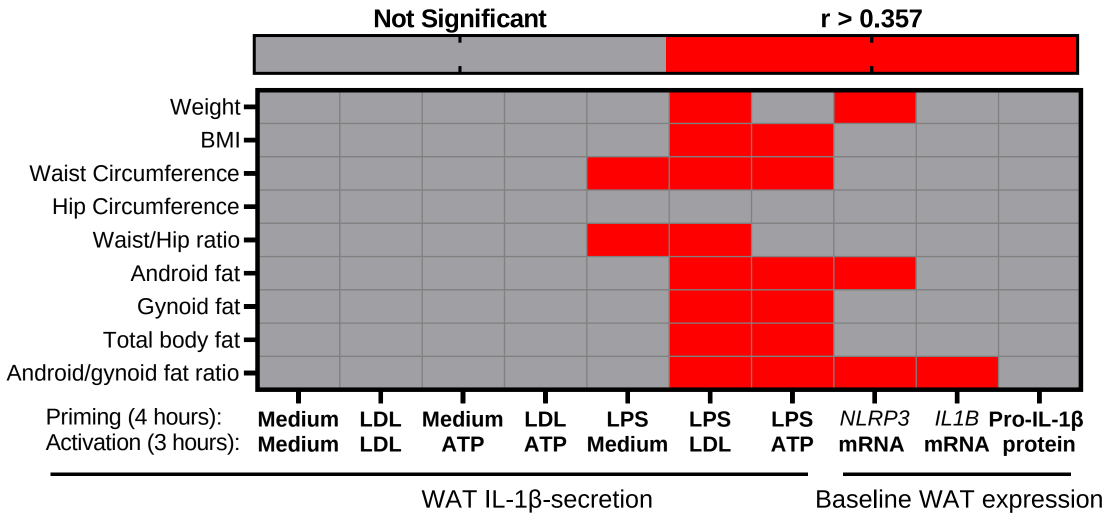

Supplemental Figure 5: Differences between subjects with low-apoB versus high-apoB in % fasting plasma phospholipid fatty acids. N=19 for low-apoB and N=20 for high-apoB for all fatty acids except for % C17:0 where N=5 for low and N=2 for high-apoB, % C16:1n-7 where N=16 for low-apoB, and % C18:3n-6 where N=18 for low and N=19 for high- apoB as the sample for one subject with low-apoB could not be measured and the named fatty acids were below detection limit of the GC-MS in some subjects.

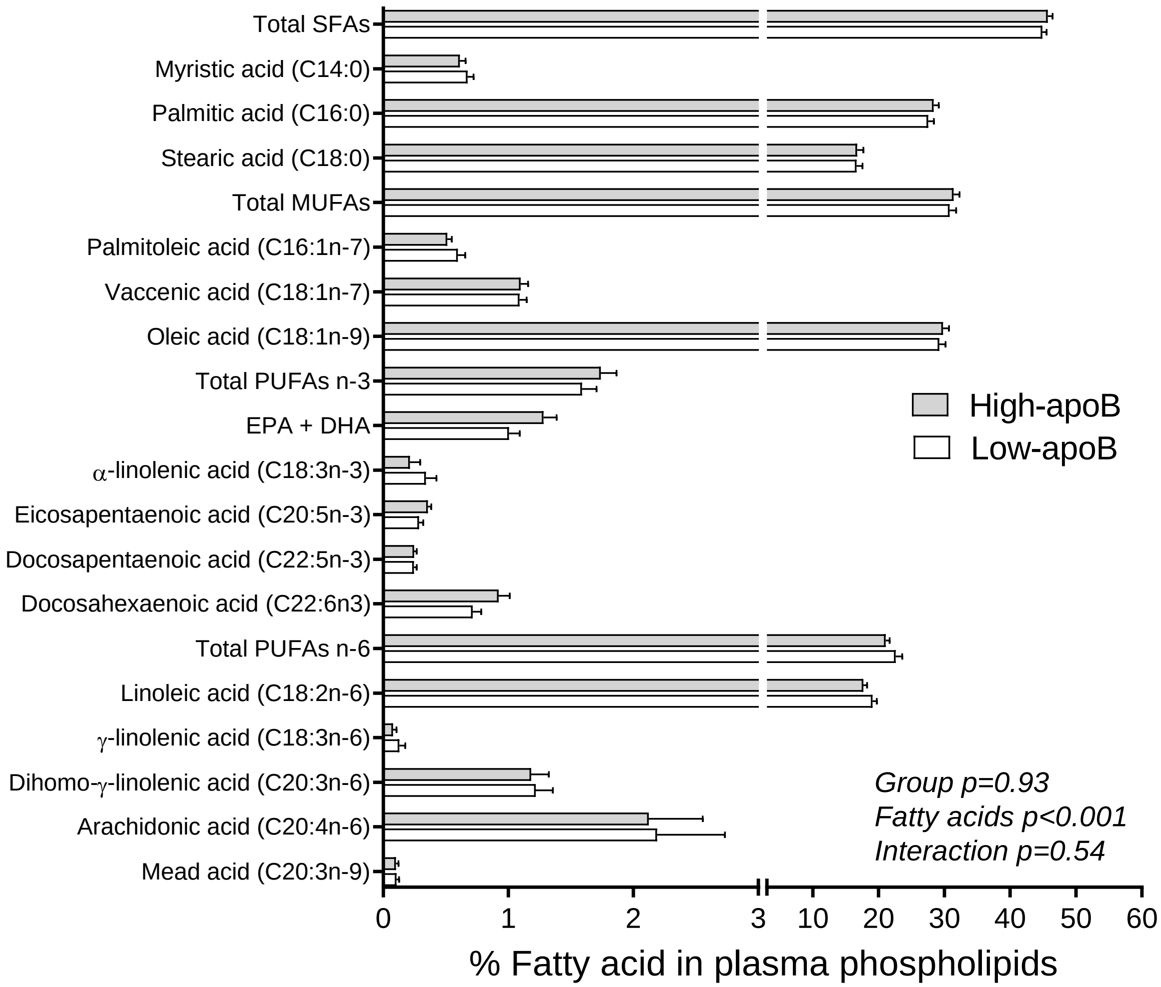

Supplemental Figure 6: Differences between subjects with low-apoB versus high-apoB in fasting baseline WAT mRNA expression of *ADIPOQ, PPARG, HMGCR, SREBP1C, SREBP2, LDLR, and CD36* normalized for *HPRT* (**A**), and of *MCP1*, *ADGRE1*, *IL10*, *NLRP3*, *CASP1*, and *IL1B* normalized for *HPRT* (**B**) analyzed by mixed-model analysis with group x gene interaction and presented as boxes with whiskers representing 10th – 90th percentile and line at the average. N=20 for low and N=20 for high plasma apoB.

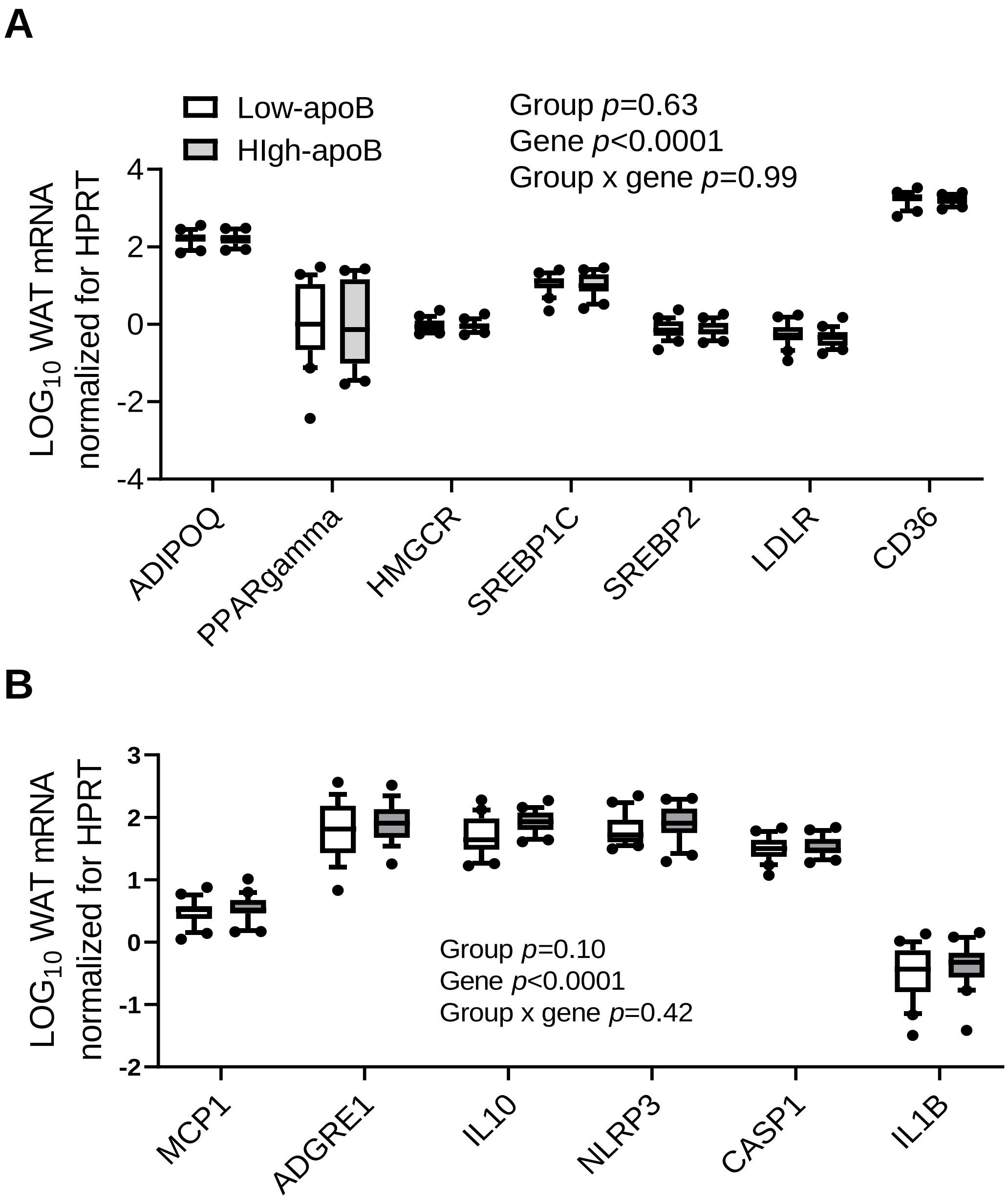

Supplemental Figure 7: Pearson correlation of fasting plasma apoB with 2nd phase $GIIS_{IVGTT}$ (**A**), total $GIIS_{IVGTT}$ (**B**), 2nd phase C-peptide $secretion_{IVGTT}$ (**C**), total C-peptide $secretion_{IVGTT}$ (**D**), insulin sensitivity as M/I_{clamp} (**E**), fasting plasma IL-1Ra (**F**), AUC_{6hr} plasma TG (**G**) and AUC_{6hr} plasma apoB48 (**H**) in women (N=27, closed circles, dotted regression line) and men (N=13, open circles, dashed regression line) except for panels **A-D** where N=11 men and panel **E** where N=12 men for missing data. Solid regression line represents pooled data for both sexes.

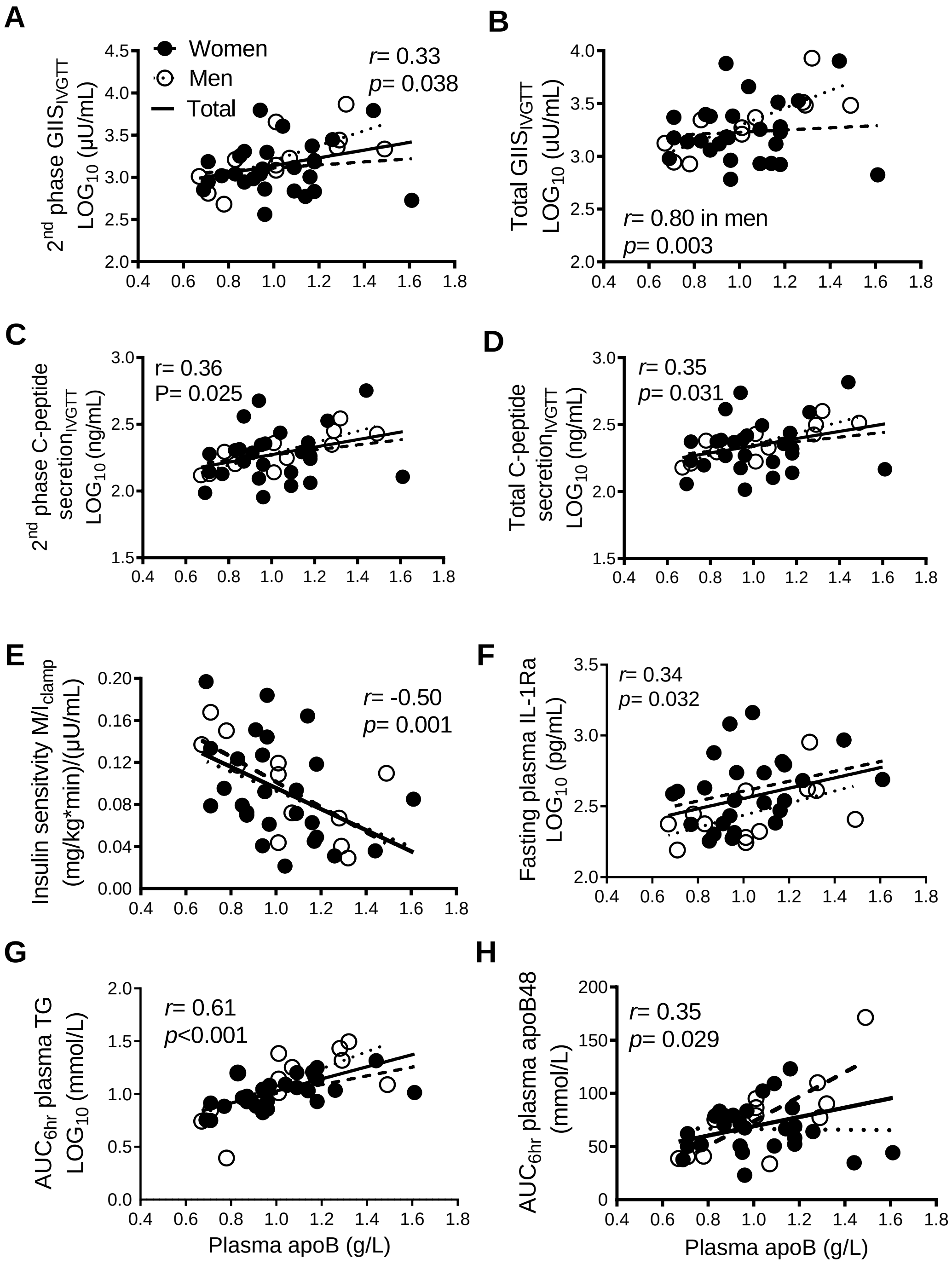

Supplemental Figure 8: Pearson correlation of fasting WAT IL-1β-secretion induced by LPS/LDL with 2nd phase $GIIS_{IVGTT}$ (**A**), total $GIIS_{IVGTT}$ (**B**), insulin sensitivity as M/I_{clamp} (**C**), AUC_{6hrs} plasma apoB48 (**D**), AUC_{6hrs} plasma TG (**E**), fasting baseline WAT mRNA expression of *ADIPOQ* (**F**) and *CD36* (**G**) normalized for *HPRT* and fasting plasma apoB (**H**), and estimated LDL size as LDL-C/apoB ratio (**I**) in subjects with low-apoB (N=15, open circles) and high-apoB (N=19, closed circles). Solid regression line represents correlation in all subjects.

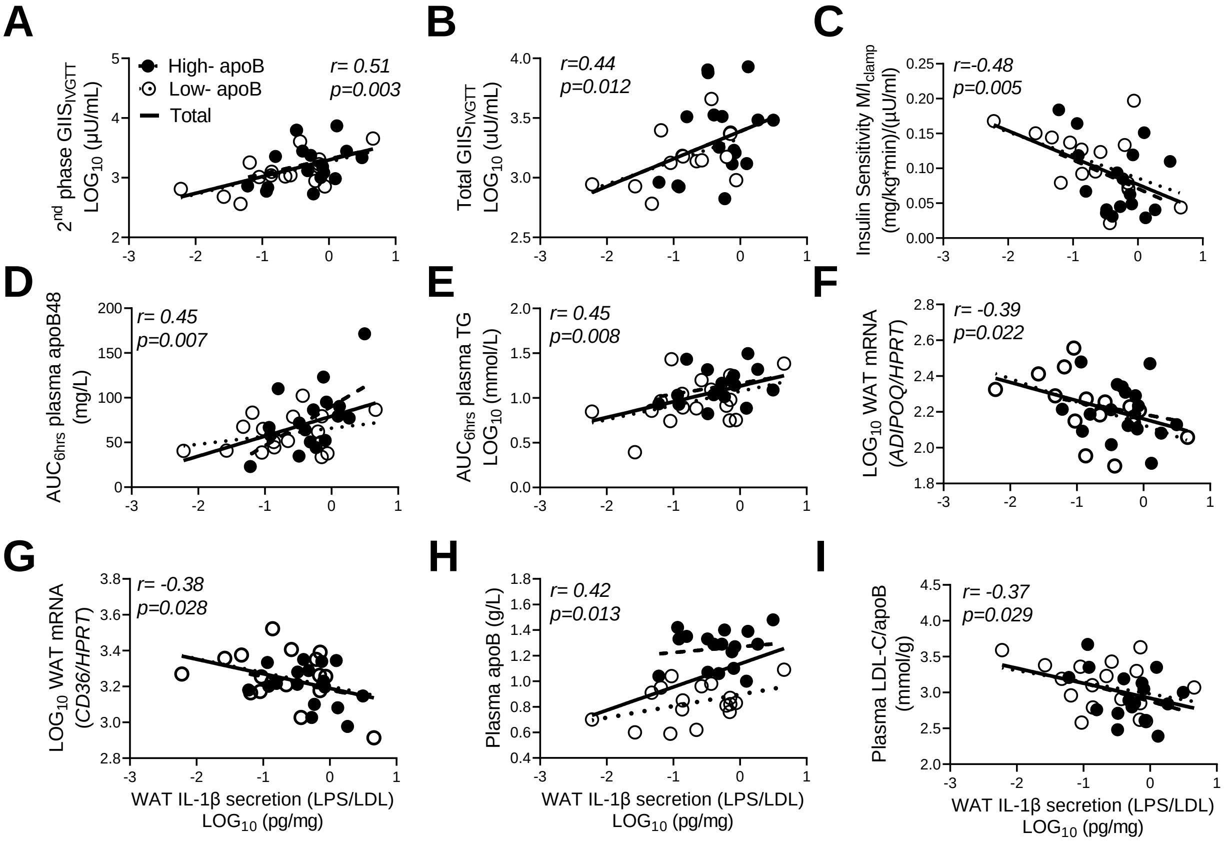

Supplemental Figure 9: Pearson correlation of fasting baseline WAT mRNA expression of *NLRP3* normalized for *HPRT* with 1st phase C-peptide secretion$_{IVGTT}$ (**A**), 2nd phase C-peptide secretion$_{IVGTT}$ (**B**), total C-peptide secretion$_{IVGTT}$ (**C**), insulin sensitivity as M/I$_{clamp}$ (**D**), total disposition index (total C-peptide$_{IVGTT}$ x M/I$_{clamp}$) (**E**), AUC$_{6hrs}$ plasma TG (**F**), fasting plasma IL-1Ra (**G**), TG (**H**), apoB/PCSK9 ratio (**I**), LDL-C/apoB ratio (**J**), and HDL-C (**K**), fasting baseline WAT mRNA expression of *PPARG* (**L**), *ADIPOQ* (**M**), C*D36* (**N**), *SREBP2* (**O**), *MCP1* (**P**), *ADGRE1* (**Q**), and *IL1B* (**R**) normalized to *HPRT*, and % fasting plasma phospholipid palmitate (**S**), arachidonate (**T**) and stearate (**U**) in subjects with low-apoB (N=20, open circles, dotted regression line) and high-apoB (N=20, closed circles, dashed regression line). Solid regression line represents pooled data for all subjects.

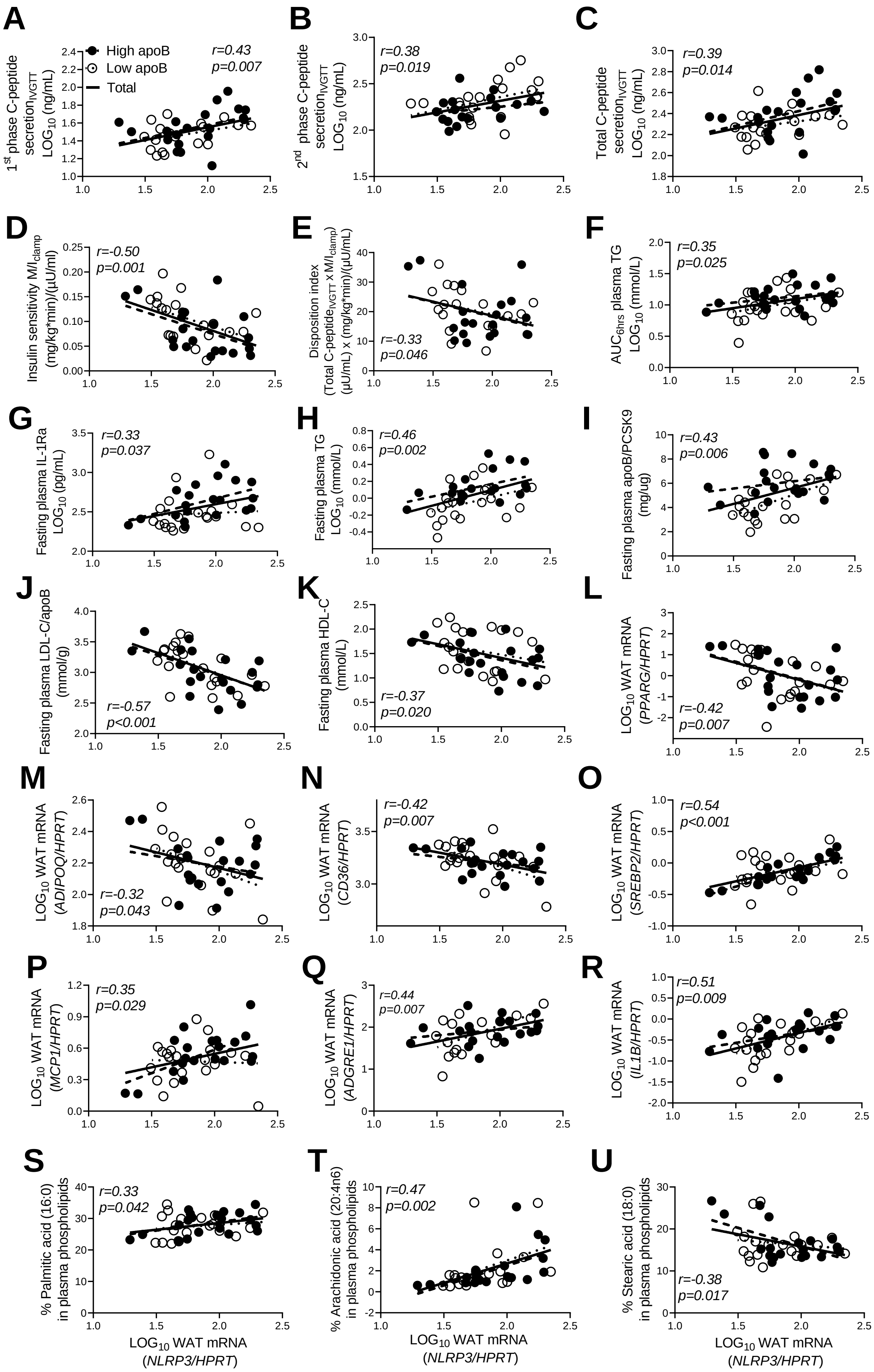

A
High apoB
Low apoB
Total
r=0.43
p=0.007
1st phase C-peptide secretion IVGTT LOG10 (ng/mL)
B
r=0.38
p=0.019
2nd phase C-peptide secretion IVGTT LOG10 (ng/mL)
C
r=0.39
p=0.014
Total C-peptide secretion IVGTT LOG10 (ng/mL)
D
r=-0.50
p=0.001
Insulin sensitivity M/I clamp (mg/kg*min)/(µU/ml)
E
r=-0.33
p=0.046
Disposition index (Total C-peptide IVGTT x M/I clamp) (µU/mL) x (mg/kg*min)/(µU/mL)
F
r=0.35
p=0.025
AUC 6hrs plasma TG LOG10 (mmol/L)
G
r=0.33
p=0.037
Fasting plasma IL-1Ra LOG10 (pg/mL)
H
r=0.46
p=0.002
Fasting plasma TG LOG10 (mmol/L)
I
r=0.43
p=0.006
Fasting plasma apoB/PCSK9 (mg/ug)
J
r=-0.57
p<0.001
Fasting plasma LDL-C/apoB (mmol/g)
K
r=-0.37
p=0.020
Fasting plasma HDL-C (mmol/L)
L
r=-0.42
p=0.007
LOG10 WAT mRNA (PPARG/HPRT)
M
r=-0.32
p=0.043
LOG10 WAT mRNA (ADIPOQ/HPRT)
N
r=-0.42
p=0.007
LOG10 WAT mRNA (CD36/HPRT)
O
r=0.54
p<0.001
LOG10 WAT mRNA (SREBP2/HPRT)
P
r=0.35
p=0.029
LOG10 WAT mRNA (MCP1/HPRT)
Q
r=0.44
p=0.007
LOG10 WAT mRNA (ADGRE1/HPRT)
R
r=0.51
p=0.009
LOG10 WAT mRNA (IL1B/HPRT)
S
r=0.33
p=0.042
% Palmitic acid (16:0) in plasma phospholipids
T
r=0.47
p=0.002
% Arachidonic acid (20:4n6) in plasma phospholipids
U
r=-0.38
p=0.017
% Stearic acid (18:0) in plasma phospholipids
LOG10 WAT mRNA (NLRP3/HPRT)
